# Epidermal Cell Dynamics Regulates Rice Lamina Joint Morphogenesis and Leaf Angle Formation through *OsZHD1* and *OsZHD2* Regulation

**DOI:** 10.1002/advs.202518691

**Published:** 2025-12-03

**Authors:** Yiru Xu, Heng Zhou, Xiaojiang Wu, Wuyu Cui, Shouling Xu, Xi He, Dan Xiang, Ming Zhou, Xiuqin Rao, Lilan Hong

**Affiliations:** ^1^ Key Laboratory of Nuclear Agricultural Sciences of Ministry of Agriculture Institute of Nuclear Agricultural Sciences College of Agriculture and Biotechnology Zhejiang University Hangzhou 310058 China; ^2^ The Advanced Seed Institute National Key Laboratory of Rice Breeding and Biology Zhejiang Provincial Key Laboratory of Crop Germplasm College of Agriculture and Biotechnology Zhejiang University Hangzhou 310058 China; ^3^ State Key Laboratory of Plant Environmental Resilience College of Life Sciences Zhejiang University Hangzhou Zhejiang 310058 China; ^4^ College of Biosystems Engineering and Food Science Zhejiang University Hangzhou 310058 China

**Keywords:** epidermis, lamina joint, organ morphogenesis, OsZHD, plant architecture

## Abstract

The lamina joint is a critical determinant of leaf angle and crop architecture. While epidermal cells play a fundamental role in organ morphogenesis, influencing the overall shape and function of plants, their impact on lamina joint morphology has been largely overlooked. A live‐imaging system for the rice lamina joint epidermis is established in this study, enabling precise tracking of cellular dynamics during leaf angle formation. It is found that asymmetric elongation between the lateral and medial edges, determined by spatial differences in the longitudinal elongation and number of epidermal cells, is a key factor in leaf angle formation. Mutations in the homeobox genes *OsZHD1* and *OsZHD2* disrupt the growth patterns of lamina joint epidermal cells, resulting in a decreased leaf angle. Epidermis‐specific restoration of *OsZHD1* expression rescues the reduced leaf angle phenotype of *oszhd1 oszhd2*, confirming the pivotal role of epidermal development in lamina joint morphogenesis. Transcriptomic analysis indicates that *OsZHD1* and *OsZHD2* regulate auxin activity, which modulates leaf angle by restricting lamina joint epidermal growth. This study underscores the significance of epidermal cells in shaping the lamina joint and elucidates the critical role of *OsZHD1* and *OsZHD2* in regulating epidermal cell behavior and leaf angle formation.

## Graphical abstract

The lamina joint determines leaf angle and plant architecture. Xu et al. establish a live‐imaging system for the rice lamina joint and reveal that asymmetric epidermal cell elongation and division between the lateral and medial edges drive leaf angle formation. OsZHD1 and OsZHD2 coordinate this process by modulating auxin activity and BR signaling.

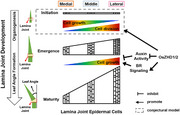

## 1 Introduction

Leaf erectness, which is determined by the leaf angle, is a critical trait for optimizing plant architecture in cereals.^[^ ^1^, ^2^ ^]^ In crops like wheat and maize, an ideal canopy structure features compact, tower‐shaped plants with erect upper leaves to enhance light penetration and more horizontal lower leaves to maximize light interception.^[^ ^3^ ^]^ Similarly, in rice (*Oryza sativa*), leaf angle directly affects the leaf area index, light interception, nitrogen utilization efficiency, and canopy aeration. A smaller leaf angle promotes efficient light capture for photosynthesis, facilitates nitrogen accumulation in leaves and its translocation to grains, and improves canopy aeration, ultimately contributing to increased yield under high‐density planting.^[^ ^4^, ^5^ ^]^ Conversely, an excessively large leaf angle can cause shading, reduce light energy use efficiency, and increase lodging risk.^[^ ^5^, ^6^, ^7^ ^]^ The establishment of leaf angle is therefore crucial. The leaf angle in rice is primarily determined by the lamina joint, which connects the leaf blade and the leaf sheath.^[^ ^8^ ^]^ The morphology of the lamina joint is governed by its cytological structures, which are regulated by both hormonal signals and environmental factors.^[^ ^5^, ^8^, ^9^ ^]^

The well‐ordered cellular organization provides the basic structure of the lamina joint. Transverse section analyses reveal that the internal tissue of the rice lamina joint primarily consists of parenchyma cells, sclerenchyma cells, vascular bundles, and aerenchyma. Parenchyma cells form the basic structural framework, while sclerenchyma tissues offer essential mechanical support. The leaf angle results from a balance between the pushing force from the expanding parenchymal cells at the adaxial side of the lamina joint and the supporting force from the structural components of the lamina joint. The vascular bundles and aerenchyma facilitate the transport of water and nutrients while ensuring air circulation.^[^ ^5^, ^8^, ^10^ ^]^

Based on the morphological features and cytological behaviors of the lamina joint, its development can be divided into six successive stages.^[^ ^8^, ^11^ ^]^ Systemic observations of the lamina joint at different developmental stages reveal distinct cytological changes. Global transcriptome analyses indicate that these cytological changes are accompanied by corresponding shifts in gene expression at each stage of lamina joint development.^[^ ^8^, ^10^ ^]^ During the early stages, active cell proliferation is supported by the expression of genes related to cell cycle regulation. In subsequent stages, the formation of different tissues such as aerenchyma and vascular bundles is driven by the upregulation of genes involved in cell wall biosynthesis and differentiation.

Altered leaf inclination can result from abnormal cell composition, disrupted cell division, or irregular cell elongation.^[^ ^12^, ^13^, ^14^ ^]^ Hormonal signals and environmental factors are crucial for regulating leaf angle by altering the cytological structures of the lamina joint.^[^ ^5^, ^9^, ^15^ ^]^ Several plant hormones, including brassinosteroids (BRs), auxins, gibberellins, and cytokinins, are involved in this regulation.^[^ ^13^, ^14^, ^16^ ^]^ Environmental changes can trigger hormone‐mediated responses that modulate cell growth at various stages of lamina joint development.^[^ ^9^ ^]^ Among these regulators, auxin biosynthesis and signaling are critical for controlling leaf inclination. Auxin has a negative regulatory role in leaf angle formation, as reduced auxin levels lead to enlarged leaf angles by promoting parenchyma cell division and elongation at the adaxial side.^[^ ^17^, ^18^, ^19^ ^]^ Treatment of the rice flag leaf lamina joint with 1‐naphthaleneacetic acid (NAA, a synthetic auxin) can reduce flag leaf angle.^[^ ^12^ ^]^ Auxin also regulates the secondary cell wall biosynthesis of sclerenchyma cells; reduced cell wall thickness in these cells leads to an exaggerated leaf angle, as they fail to adequately support the leaf, resulting in bending away from the vertical axis.^[^ ^12^ ^]^

Although previous studies have demonstrated the involvement of multiple factors in lamina joint development and systematically revealed how the growth dynamics of different tissues in the lamina joint determine leaf angle, there remains a notable lack of cytological observations on the epidermal cells of the lamina joint. The development of the epidermis is critically important for controlling organ morphology. As the outermost tissue, the epidermis provides structural integrity; its rigidity and arrangement significantly contribute to the overall architecture of the plant, helping to maintain its shape and stability.^[^ ^20^, ^21^ ^]^ Furthermore, epidermal cells can either limit or drive the growth of internal tissue cells within organs.^[^ ^22^ ^]^ The epidermis also plays a key role in regulating organ growth through various plant hormones, such as BRs and ethylene, serving as the primary site for their action.^[^ ^23^, ^24^ ^]^

**Figure 1:**
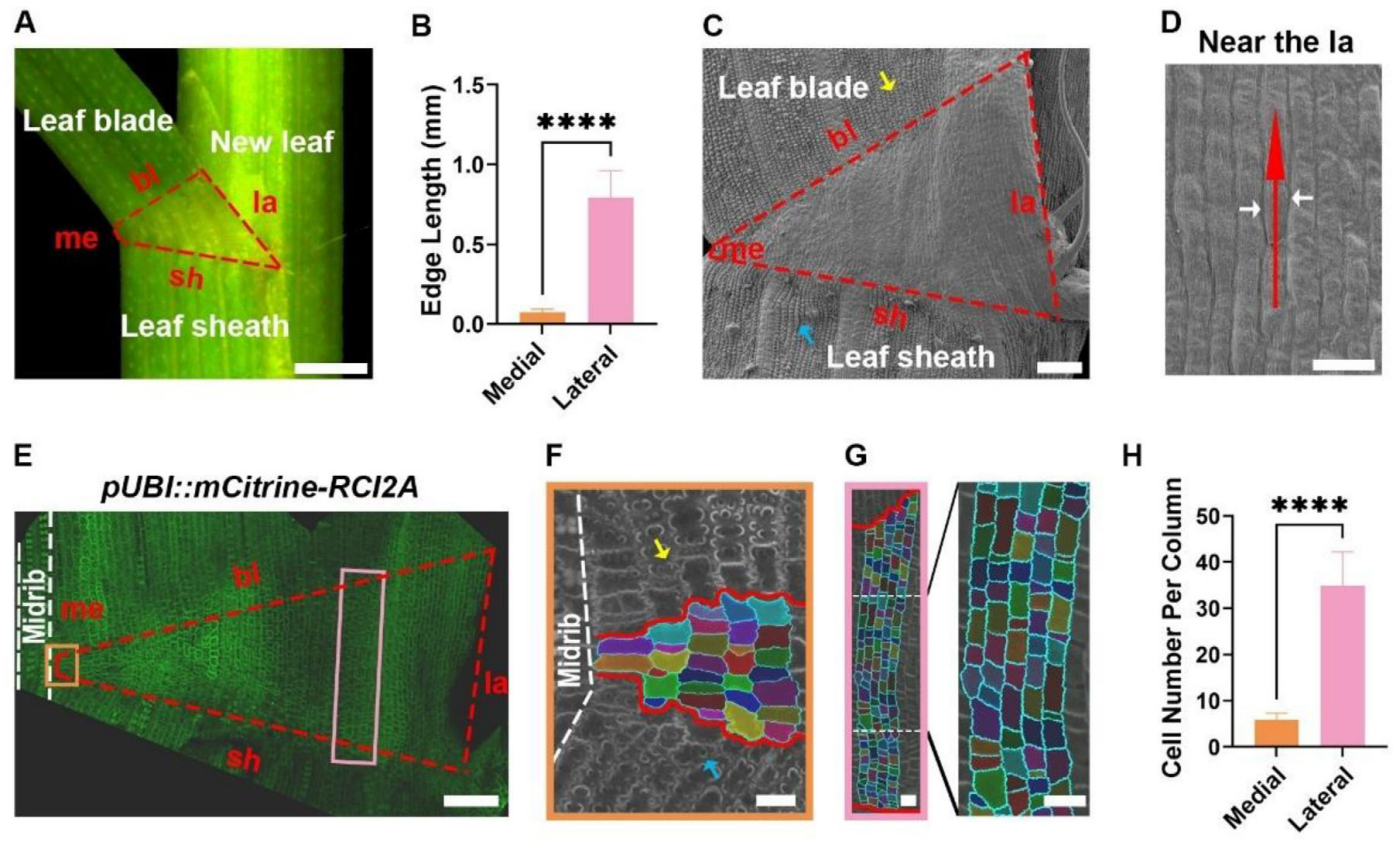
The lateral edge of the rice lamina joint contains more epidermal cells, resulting in a greater length compared to the medial edge. A) Morphological diagram of the lamina joint epidermis. Scale bar, 0.5 mm. B) Lengths of the lateral edge and the medial edge of the lamina joint from the second complete leaf of 4‐week‐old seedlings. Data are presented as mean ± SD (n = 10). Statistical significance is determined using Student's *t*‐test (^****^ *P* < 0.0001). C,D) Scanning electron micrographs of the lamina joint epidermis. (C) shows the entire epidermis of the lamina joint under scanning electron microscopy. The red dashed lines outline the lamina joint area. The yellow and blue arrowheads indicate papillae attached to the leaf blade and leaf sheath, respectively. Scale bar, 100 µm. (D) shows cells near the lateral edge, with the red arrow pointing to the cell files, while the white arrows indicate borders between cell files. Scale bar, 25 µm. E–G) Confocal images of the lamina joint using a plasma membrane reporter (*pUBI::RCI2A‐mCitrine*). (E) shows the entire lamina joint captured by laser confocal microscopy. The white dashed lines highlight the midrib region adjacent to the medial edge. Scale bar, 100 µm. (F) is a magnified view of the orange box in (E). The region between the solid red lines is the lamina joint. Yellow and blue arrowheads denote papillae on the leaf blade and leaf sheath, respectively. Scale bar, 20 µm. (G) The left panel is a magnified view of the pink box in (E), also with the lamina joint outlined by solid red lines. The right panel is a further magnification of the boxed area in the left panel. Scale bars, 20 µm. H) Comparison of epidermal cell number between the medial and lateral edges. Data are presented as mean ± SD (n = 10). Statistical analysis is performed using Student's *t*‐test (^****^ *P* < 0.0001).

Here we concentrated our observations on the epidermal morphology of the lamina joint. The epidermis of the mature lamina joint resembles an irregular quadrilateral when viewed from the side (facing the leaf angle). To investigate the lamina joint morphogenesis in detail, we established a nomenclature for its four edges: the edge closest to the new leaf is the lateral edge, while the edge farthest from the new leaf is the medial edge; the blade edge is at the junction of the lamina joint and the leaf blade, and the sheath edge is at the junction of the lamina joint and the leaf sheath (**Figure** **1**A). We hypothesize that epidermal development plays a critical role in leaf angle formation, and we utilized live imaging and image processing techniques to track the growth and division behavior of rice lamina joint epidermal cells. Based on this hypothesis, we predict that abnormalities in the development of the lamina joint epidermis will influence the size of the leaf angle.

## 2 Results

### 2.1 The Lateral Edges have More Epidermal Cells than the Medial Edges in Rice Lamina Joint

Organ morphogenesis in plants is largely confined by epidermal cells.^[^ ^20^, ^21^, ^22^, ^23^, ^24^ ^]^ However, very little is known about the role of epidermal cells in lamina joint morphogenesis in grass. We aimed to address this issue in rice and imaged the lamina joint of the second complete leaf of 4‐week‐old rice seedlings using scanning electron microscopy (SEM), in order to obtain an overview of the morphology of epidermal cells in the rice lamina joint (Figure 1C,D). Our observations revealed that the surface of the leaf blade and sheath epidermal cells was sprinkled with papillae, whereas the surface of the lamina joint epidermal cells was relatively smooth and lacked papillae. This difference in the abundance of papillae is a prominent feature distinguishing lamina joint epidermal cells from those of the leaf blade and leaf sheath (Figure 1C). Although neatly arranged cell files can be clearly seen under the SEM, it remains challenging to differentiate each cell individually (Figure 1D).

To clearly visualize the epidermal cells of the lamina joint, we created a transgenic rice line with a plasma membrane reporter (*pUBI::mCitrine‐RCI2A*) and performed static imaging on dissected lamina joints of the second complete leaf from 4‐week‐old reporter line seedlings. Using confocal laser scanning microscopy (CLSM), we conducted z‐stack scanning to capture 3D images of the lamina joint. MorphoGraphx software was employed to specifically detect the fluorescence signals of the epidermal cells and segment the epidermal cells (Figure 1E). Our analysis revealed an obvious difference in the number of epidermal cells between the medial edge and lateral edge of lamina joints (Figure 1F–H): the medial edge contained only a few cells (Figure 1F), while the lateral edge had dozens (Figure 1G). Given that the lateral edge is longer than the medial edge at this developmental stage (Figure 1B), and there is no significant difference (Figure S1A,B, Supporting Information) in the area of lamina joint epidermal cells, we suggest that the larger number of cells along the lateral edge is a key factor contributing to this difference.

### 2.2 The Increased Leaf Angle Results from the Asymmetric Elongation between the Lateral and Medial Edges

Based on previous reports, the developmental processes of the rice second complete leaf lamina joint are divided into six successive stages (Zhou et al., 2017; Wang et al., 2020; Liu et al., 2024). Stage 1 to 3 are defined as the stages of organogenesis, during which the lamina joint is enveloped by the sheath of the previous leaf. Stages 4 to 6 correspond to the stages of leaf angle formation, during which the lamina joint is exposed to sunlight and begins to bend, forming a curvature. To elucidate the controlling mechanism of leaf angles, we focused on observing the epidermal morphology changes of lamina joints during the process of leaf angle formation, spanning from the emergence of the lamina joint (the beginning of stage 4, when the seedling is 15‐day‐old and the lamina joint of the second complete leaf begins to be exposed to sunlight) to the maturity of the lamina joint (the end of stage 6, when the seedling is 27‐day‐old and the leaf angle of the second complete leaf reaches its maximum). The transverse size of the lamina joint reaches its maximum at stage 4 (Zhou et al., 2017). Our findings also showed that from the emergence to maturity, the lamina joint mainly changes the length of the lateral edge, suggesting that the increase in the length of the lateral edge is the primary cause of the lamina joint's morphological changes (Figure S2, Supporting Information).

**Figure 2:**
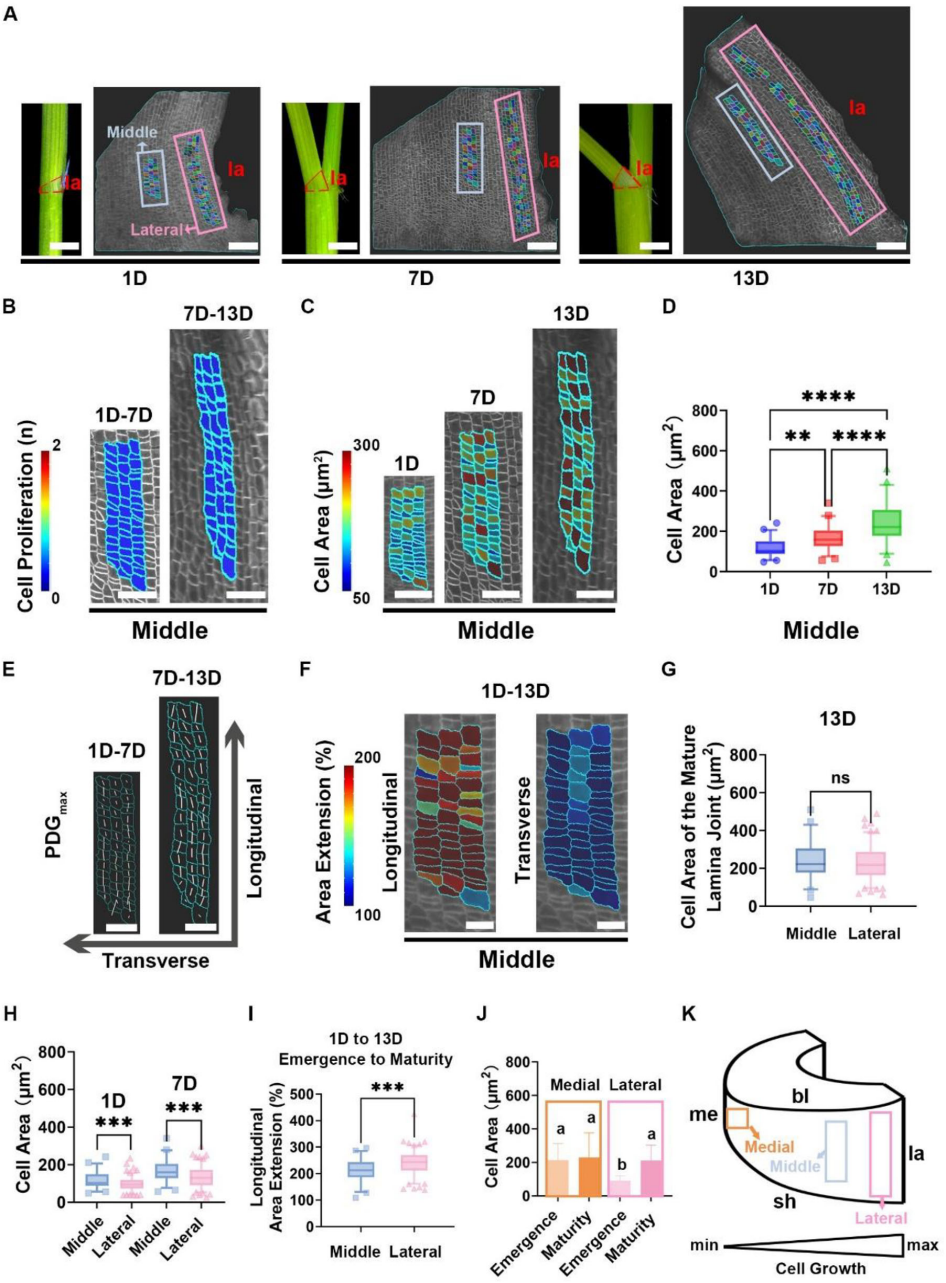
The epidermal cells of the lamina joint elongate along the cell files during leaf angle formation. A) Real‐time imaging of the lamina joint growth. The morphology of the lamina joint was observed on the 1D, 7D, and 13D, and the epidermal cells were visualized under a confocal laser scanning microscope at these corresponding time points. Cells derived from the same mother cell at the starting time point were labeled with the same color. The red dashed lines outline the lamina joint in the stereoscope images on the left, while the blue and pink boxes mark the middle and lateral regions in the confocal images on the right, respectively. Scale bars: 1 mm for stereoscope images, 100 µm for confocal images. la, the lateral edge. B) Cell proliferation in the middle region that divided during each growth interval (shown at the later time point). Scale bars, 50 µm. C) Heatmaps of cell area in the middle region. Scale bars, 50 µm. D) Boxplots showing the epidermal cell area as in (C). n = 50 cells. Statistical analysis is conducted using one‐way ANOVA followed by Tukey's post‐hoc test (^**^ *P* < 0.01, ^****^ *P* < 0.0001). E) The directions of maximal growth (PDG_max_; PDG, principal direction of growth) for epidermal cells are indicated by white lines (shown at the later time point). The longitudinal axis is parallel to the axis of the cell files, while the transverse axis is perpendicular to it. Scale bars, 40 µm. F) Heatmap of area extension (%) in the longitudinal and transverse directions from 1D to 13D of observation (shown at 1D). Scale bars, 20 µm. G) Boxplots of cell area for the middle and lateral regions. n = 134 cells for the lateral region, n = 50 cells for the middle region. Statistical analysis is conducted using Student's *t*‐test (ns = no significance). H) Boxplots of cell area for the middle and lateral regions at 1D and 7D. n = 134 cells for the lateral region, n = 50 cells for the middle region. Statistical analysis is conducted using Student's *t*‐test (^***^ *P* < 0.001). I) Boxplots of longitudinal cell area extension (%) from 1D to 13D. n = 134 cells for the lateral region, n = 50 cells for the middle region. Statistical analysis is conducted using Student's *t*‐test (^***^ *P* < 0.001). J) Statistical analysis of epidermal cell area. Data are presented as mean ± SD. n = 102 cells on the medial region at the emergence stage, n = 68 cells on the medial region at the maturity stage, n = 100 cells on the lateral region at the emergence stage, n = 86 cells on the lateral region at the maturity stage. Statistical analysis is conducted using one‐way ANOVA followed by Tukey's post‐hoc test. Different letters indicate significant differences (*P* < 0.05). K) 3D schematic diagram of the lamina joint. The orange, blue, and pink boxes represent the medial, middle, and lateral regions, respectively. Spatial differences in cell elongation are observed across different regions, with the growth rate gradually increasing from the medial region to the lateral region.

To explore how the spatiotemporal behaviors of lamina joint epidermal cells impact the leaf angle size, we live‐imaged and tracked the dynamics of lamina joint morphogenesis on the cellular level during the formation of the leaf angle using the *pUBI::mCitrine‐RCI2A* seedlings. The lamina joint epidermal cells of the second complete leaf were imaged at three time points from the emergence to maturity: the 1st day (1D), the 7th day (7D), and the 13th day (13D). On the 1st day, the lamina joint was just exposed to sunlight and began forming the leaf angle. By the 7th day, the leaf angle had reached half of its mature size. By the 13th day, the leaf angle had approached its maximum size (**Figure** **2**A). Due to the technical limitations of live imaging in rice lamina joint, such as the low signal‐noise ratio of fluorescent signals resulting from the strong autofluorescence in rice tissue, it was difficult to capture the growth dynamics of all the epidermal cells. We selected cells from two representative regions on the epidermis for analysis: the region between the medial and lateral edge of the lamina joint (the middle region) and the region near the lateral edge of the lamina joint (the lateral region) (Figure 2A). Cell behaviors in these two regions were analyzed using the MorphoGraphx software. During the formation of the leaf angle, the epidermal cells of the lamina joint did not undergo cell division (Figure 2B; Figure S3A, Supporting Information). As the leaf angle gradually increased, epidermal cells in both the middle and lateral regions exhibited a significant increase in cell area (Figure 2C,D; Figure S3B,C, Supporting Information). The epidermal cells were neatly arranged in files from 1D to 13D. We defined the direction along the cell files as longitudinal and the direction perpendicular to the cell files as transverse. We quantified the cell growth along the longitudinal direction and the transverse direction. The epidermal cells primarily elongated in the longitudinal direction, with almost no growth in the transverse direction (Figure 2E,F; Figure S3D,E, Supporting Information). In 13D, with the leaf angle reaching its mature size, there was no significant difference in cell areas between different regions (Figure 2G).

Although no significant difference was observed in the area of epidermal cells in different regions after the maturation of the lamina joint, we noted that, during the early stages of leaf angle formation, the cell area in the lateral region was smaller than that in the middle region (Figure 2H). This was consistent with the observation that the growth rate of cells in the lateral region was faster than in the middle region (Figure 2I). Based on these findings, we speculated that there are spatial differences in the elongation of epidermal cells of the lamina joint. The growth rate of epidermal cell area near the medial edge may be slower than that on the lateral edge, as suggested by the above‐mentioned hypothesis. However, due to the limited scanning depth of confocal laser microscopy, it is challenging to dynamically image the epidermal cells on the medial edge. Therefore, we imaged the dissected lamina joint at both the emergence and maturity stages. As anticipated, we found that during the emergence stage, the cell area on the medial edge of the lamina joint was larger than that on the lateral edge, and the cell area on the medial edge did not increase as the lamina joint matured. In contrast, epidermal cells on the lateral edge had smaller areas during the early stages of the leaf angle formation, and their area significantly increased as the leaf angle increased (Figure 2J). These findings highlight the spatial differences in cell elongation at different positions of the lamina joint epidermis (Figure 2K).

Considering that during leaf angle formation, the epidermal cells of the lamina joint cease division and mainly elongate along the direction of the cell file, we conclude that this longitudinal elongation results in an increase in the overall lengths of the lamina joint epidermis in the longitudinal direction. Due to the significantly larger number and greater growth ratio of epidermal cells along the lateral edge compared to the medial edge, the lateral edge undergoes more elongation than the medial edge, leading to dramatic morphological changes in the lamina joint. This asymmetric elongation between the lateral and medial edges is the key contributing factor to the formation of the leaf angle.

### 2.3 Mutants Lacking OsZHD1 and OsZHD2 Exhibit Small Flag Leaf Angles

**Figure 3:**
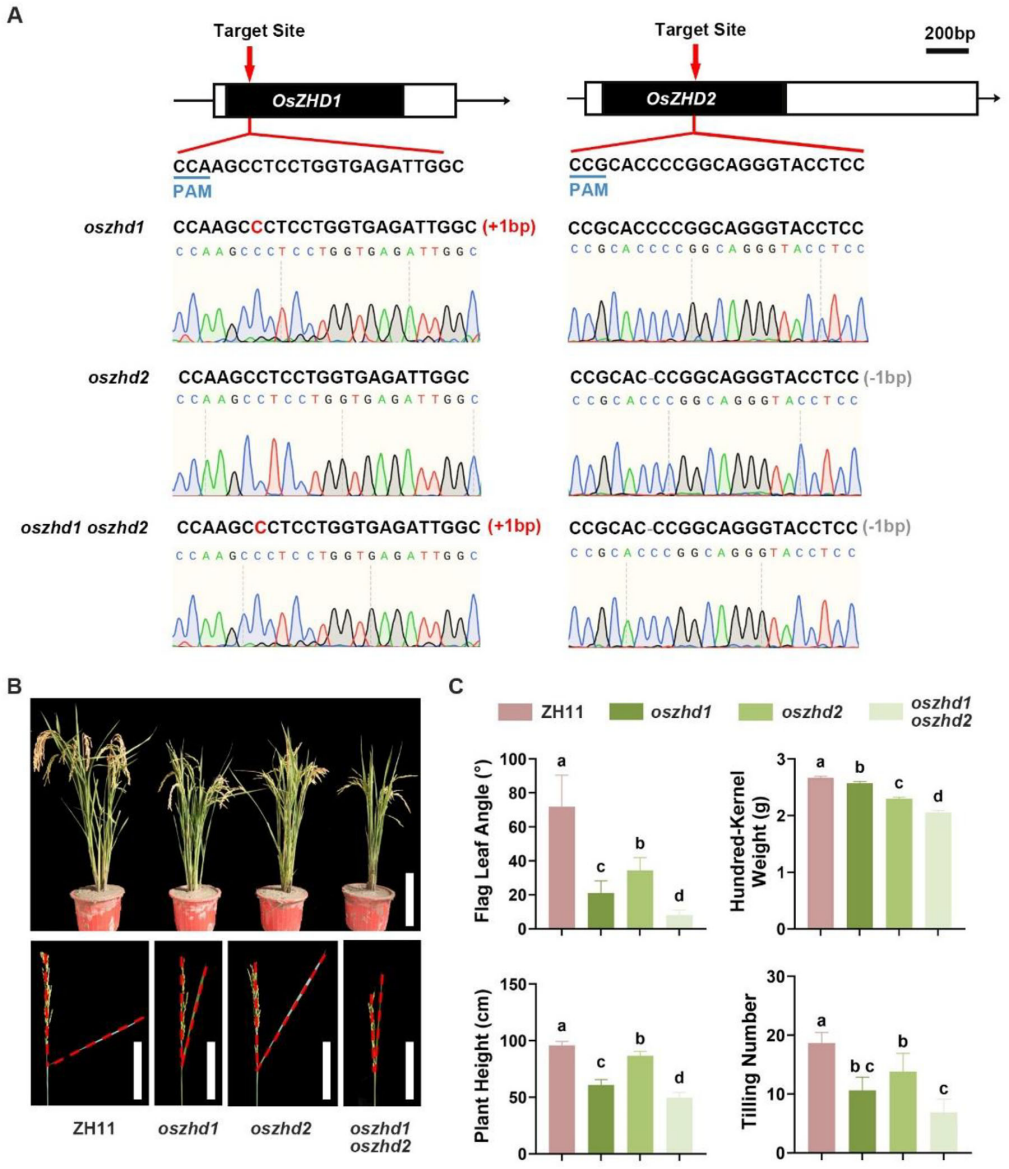
*OsZHD1* or *OsZHD2* knockout reduces leaf angle. A) Target sites and mutated sequences of *OsZHD1* and *OsZHD2*. Black boxes represent exons, while arrowheads indicate the target sites of the sgRNAs. Blue underlines mark the PAM sequences. B) Growth and flag leaf angle phenotypes of ZH11, *oszhd1*, *oszhd2*, and *oszhd1 oszhd2* at the ripening stage. The red dashed lines outline the angle between the flag leaves and the stems. Scale bars: 20 cm (upper panel); 10 cm (lower panels). C) Statistical analysis of agronomic traits of ZH11, *oszhd1*, *oszhd2*, and *oszhd1 oszhd2*. Data are presented as mean ± SD (n ≥ 10 for flag leaf angle, plant height, and tilling number; n = 5 for hundred‐kernel weight). Statistical analysis is conducted using one‐way ANOVA followed by Tukey's post‐hoc test. Different letters indicate significant differences (*P* < 0.05).

To investigate the molecular mechanisms regulating the patterns of lamina joint epidermal development and leaf inclination, we screened RNA‐seq databases covering different stages of lamina joint development.^[^ ^10^ ^]^ Our analysis revealed that the ZF‐HD family transcription factor OsZHD1 and its closest homolog OsZHD2 were potentially involved in lamina joint epidermal development. *OsZHD1* and *OsZHD2* are expressed throughout the entire stages of lamina joint development^[^ ^8^, ^10^ ^]^ (Figure S4, Supporting Information), and can significantly affect the rice architecture.^[^ ^25^ ^]^ Notably, overexpression of *OsZHD1* induces drooping leaf in rice. Both *OsZHD1* and *OsZHD2* have been demonstrated to regulate the size of bulliform cells in rice, indicating these two genes may be associated with epidermis development. Previous research also reported that OsZHD1 and OsZHD2 regulate the cellular behavior of different organs, such as roots and flowers, by participating in different hormone pathways.^[^ ^26^, ^27^ ^]^ To further investigate the role of OsZHD1 and OsZHD2 in modulating epidermal cell behaviors and leaf angle formation, we generated *oszhd1*, *oszhd2*, and *oszhd1 oszhd2* knockout lines using CRISPR/Cas9 technology (**Figure** **3**A; Table S1, Supporting Information). We observed significantly decreased flag leaf angles in both *oszhd1* (21.00° ± 7.18°) and *oszhd2* mutants (34.25° ± 7.66°), with a more pronounced flag leaf angle phenotype in the *oszhd1 oszhd2* double mutant lines (8.00 ± 2.99°) compared to ZH11 (71.83° ± 18.59°) (Figure 3B,C), suggesting essential roles for *OsZHD1* and *OsZHD2* in regulating rice leaf angles. In addition, compared to the WT, mutant plants also exhibited other agronomic phenotypes such as reduced tillering, shorter plant height and smaller grain size (Figure 3C; Figure S5, Supporting Information).

### 2.4 OsZHD1 and OsZHD2 Affect Cell Proliferation and Elongation in the Epidermal Cells of Rice Lamina Joint

**Figure 4:**
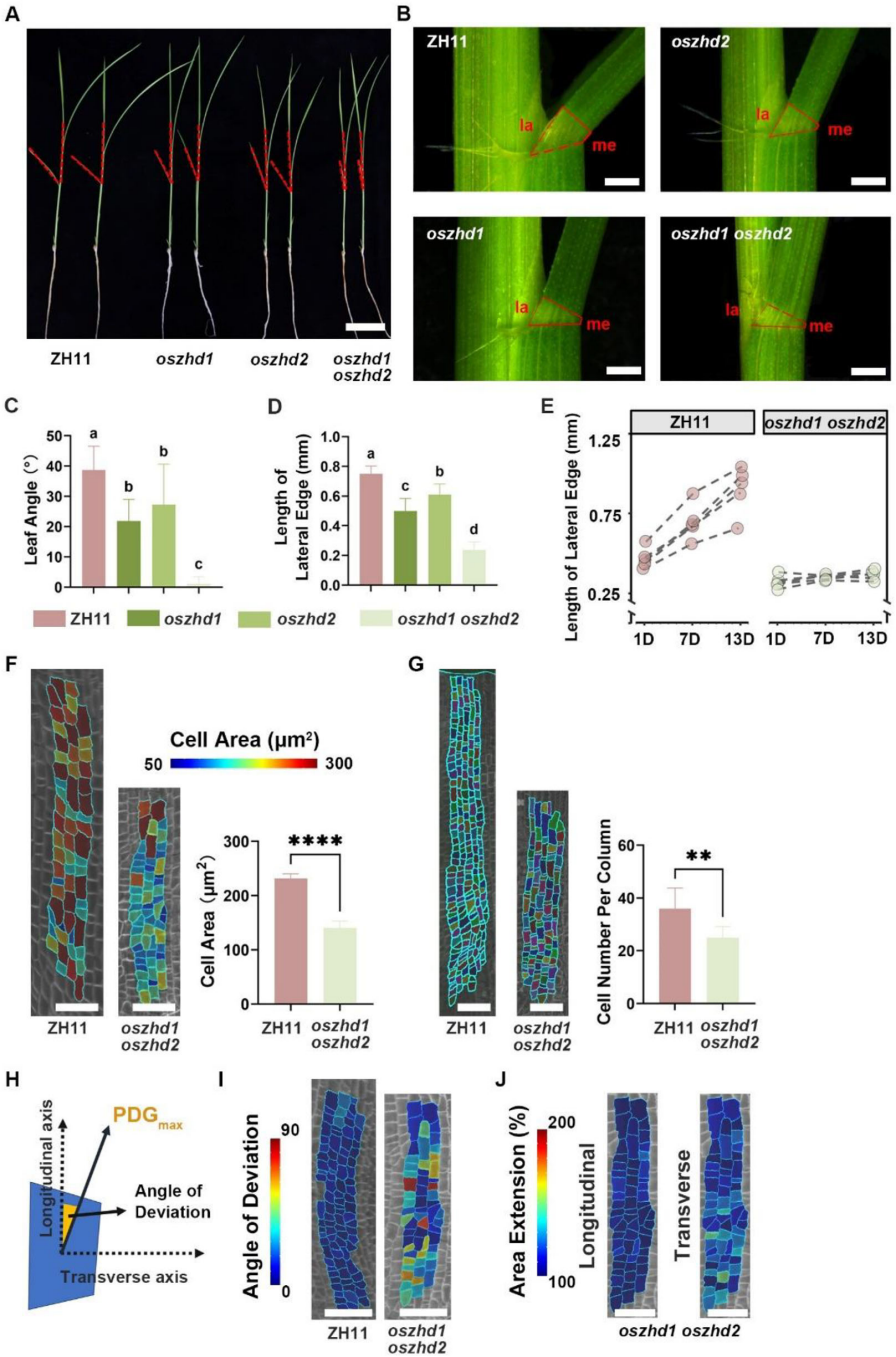
OsZHD1 and OsZHD2 affect the proliferation and elongation of epidermal cells in rice lamina joint. A) Gross morphology of 4‐week‐old seedlings of ZH11, *oszhd1*, *oszhd2*, and *oszhd1 oszhd2*. The angle outlined by the red dashed lines represents the leaf angle of the second complete leaf. Scale bar, 5 cm. B) Side views of the mature lamina joints from the second complete leaves of the seedlings shown in (A). la: lateral edge, me: medial edge. Scale bars, 0.5 mm. C) Measurement of the leaf angle of the seedlings shown in (A). Data are presented as mean ± SD (n ≥ 8). Statistical analysis is conducted using one‐way ANOVA followed by Tukey's post‐hoc test. Different letters indicate significant differences (*P* < 0.05). D) Measurement of the length of the lateral edge shown in (B). Data are presented as mean ± SD (n ≥ 8). Statistical analysis is conducted using one‐way ANOVA followed by Tukey's post‐hoc test. Different letters indicate significant differences (*P* < 0.05). E) Dynamic changes in the lateral edge length of ZH11 and *oszhd1 oszhd2*, n = 6. F) Heatmap of epidermal cell area of mature ZH11 and *oszhd1 oszhd2* lamina joints. Scale bars, 50 µm. Data are presented as mean ± SD (n = 505 cells for ZH11, n = 101 cells for *oszhd1 oszhd2*). Statistical analysis is performed using Student's *t*‐test (^****^ *P* < 0.0001). G) Epidermal cells near the fifth vascular bundle. Scale bars, 50 µm. Data are presented as mean ± SD (n = 8). Statistical analysis is performed using Student's *t*‐test (^**^ *P* < 0.01). H) Schematic diagram depicting the calculation of the angle of deviation between the PDG_max_ tensor and the longitudinal axis to obtain a quantitative measure of deviation. I) Heatmaps showing the angle of deviation of the PDG_max_ tensor from the lamina joint longitudinal axis for epidermal cells of ZH11 and *oszhd1 oszhd2*. Scale bars, 50 µm. J) Heatmap of area extension (%) of cells in *oszhd1 oszhd2* in both longitudinal and transverse directions from 1D to 13D of observation (shown at 1D). Scale bars, 20 µm.

Consistent with the observed phenotypes in flag leaf angles, the mutants also exhibited significantly smaller leaf angles at seedling stages (**Figure** **4**A,C). To elucidate the mechanisms by which *OsZHD1* and *OsZHD2* affect epidermal cell behavior and leaf angle formation, we analyze the morphology of the leaf lamina joints of the second complete leaves from 4‐week‐old seedlings (Figure 4B). We imaged them under a stereomicroscope and found that in the *oszhd1* and *oszhd2*, the total length of the lamina joint on the lateral edge was smaller than that in the WT, with *oszhd1 oszhd2* further exacerbating this difference (Figure 4D). However, no significant differences were observed in the lengths of the blade edges, sheath edges, and medial edges of the lamina joints between the different genotypes (Figure S6, Supporting Information).

Due to the more pronounced leaf angle phenotypes in the double mutant, we focused on *oszhd1 oszhd2* for subsequent morphological analysis. We tracked the development of the lamina joints in WT and *oszhd1 oszhd2* and found that the growth rate of the lateral edge of the lamina joint in WT was significantly faster than that in *oszhd1 oszhd2* (Figure 4E). We introduced the plasma membrane reporter (*pUBI::mCitrine‐RCI2A*) into *oszhd1 oszhd2*. Imaging observations of mature lamina joint epidermal cells under CLSM revealed that the area of lamina joint epidermal cells in *oszhd1 oszhd2* was smaller than that of the WT. The epidermal cell area of *oszhd1 oszhd2* is ≈60% of that of the WT (Figure 4F). Additionally, fewer cells were observed in the *oszhd1 oszhd2* lamina joint epidermis. There were ≈36 cells per column near the fifth vascular bundle (from the lateral edge) of the WT lamina joint, compared to only 25 cells in the counterpart region of *oszhd1 oszhd2* lamina joint (Figure 4G). While epidermal cells primarily elongated along the longitudinal direction in the WT lamina joint, the growth direction of epidermal cells in the *oszhd1 oszhd2* lamina joint was more misoriented (Figure 4H,I). In addition, the lamina joint epidermal cells in *oszhd1 oszhd2* showed low growth rates along either the longitudinal or the transverse directions (Figure 4J, compared with Figure 2F).

In summary, the mutation of *OsZHD1* and *OsZHD2* disrupts the growth patterns of epidermal cells in the lamina joint. Our results indicate that OsZHD1 and OsZHD2 may act as positive regulators of leaf angle determination by activating the proliferation and elongation of lateral epidermal cells at the lamina joint. They also regulate the direction of cell growth.

### 2.5 Restoring *OsZHD1* Expression in the Epidermis Regulates Lamina Joint Morphology by Increasing both the Number and Size of Epidermal Cells

**Figure 5:**
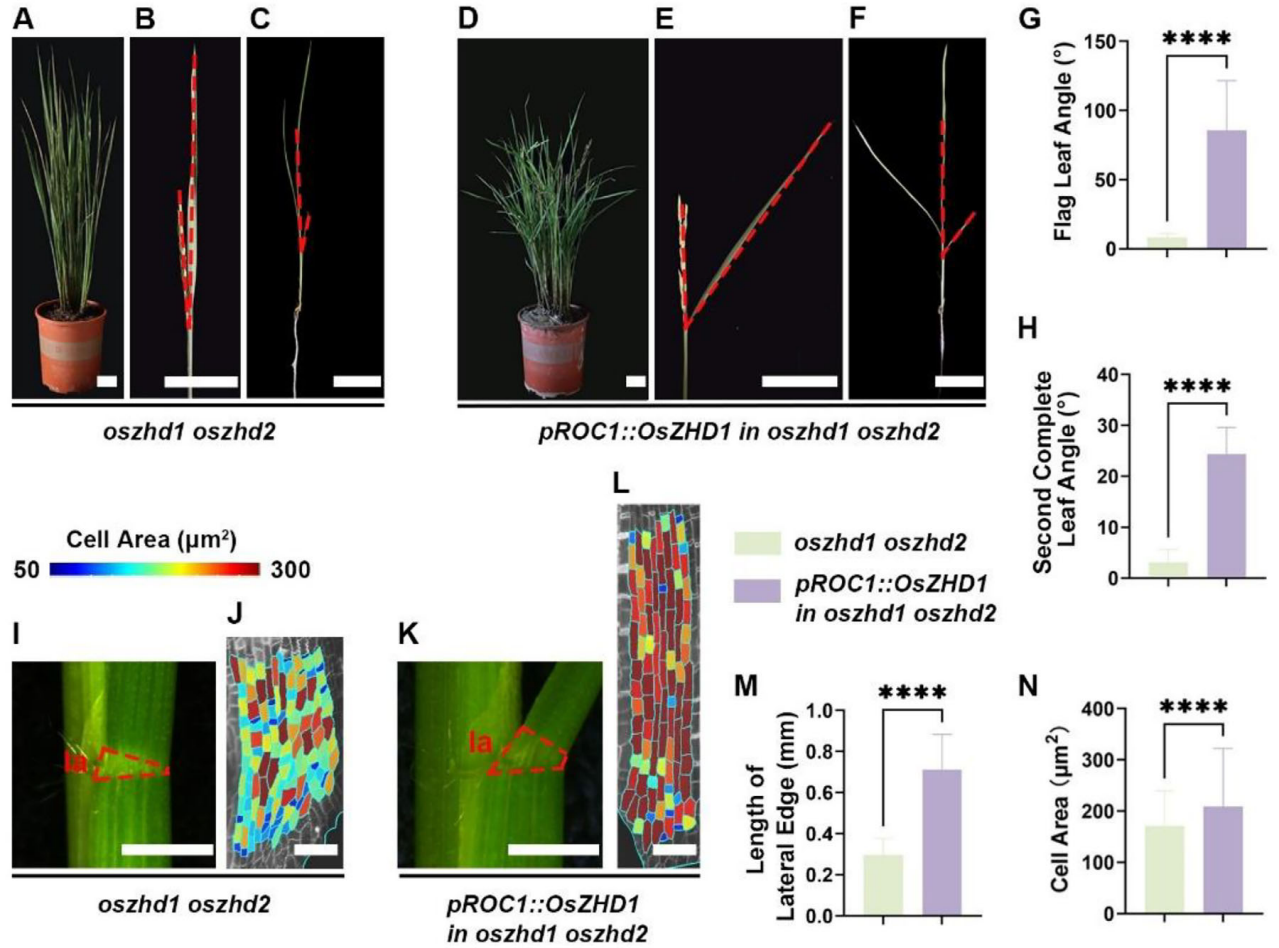
Restoring *OsZHD1* expression in the epidermis rescues the reduced leaf angle phenotype in *oszhd1 oszhd2*. A–C) *oszhd1 oszhd2* exhibits a reduced leaf angle phenotype. (A) Growth phenotype at the ripening stage. (B) Flag leaf angle phenotype at the ripening stage. (C) Gross morphology of a 4‐week‐old seedling. The angle between the red dashed lines represents the leaf angle. Scale bars, 5 cm. D–F) *pROC1::OsZHD1* rescues the reduced leaf angle phenotype in *oszhd1 oszhd2*. (D) Growth phenotype at the ripening stage. (E) Flag leaf angle phenotype at the ripening stage. (F) Gross morphology of a 4‐week‐old seedling. The angle between the red dashed lines represents the leaf angle. Scale bars, 5 cm. G) Statistical analysis of flag leaf angles at the ripening stage. Data are presented as mean ± SD (n ≥ 10). Statistical analysis is performed using Student's *t*‐test (^****^ *P* < 0.0001). H) Measurement of the second complete leaf angles from 4‐week‐old seedlings. Data are presented as mean ± SD (n = 10). Statistical analysis was performed using Student's *t*‐test (^****^ *P* < 0.0001). I) Side view of the mature lamina joint from the second complete leaf of 4‐week‐old *oszhd1 oszhd2* seedlings. The red dashed lines outline the lamina joint. la: lateral edge. Scale bar, 1 mm. J) Heatmap of the mature epidermal cell area in *oszhd1 oszhd2* as shown in (I). Scale bar, 50 µm. K) Side view of the mature lamina joint from the second complete leaf of 4‐week‐old *pROC1::OsZHD1* in *oszhd1 oszhd2* seedlings. The red dashed lines outline the lamina joint. la: lateral edge. Scale bar, 1 mm. L) Heatmap of the mature epidermal cell area of *pROC1::OsZHD1* in *oszhd1 oszhd2* as shown in (K). Scale bar, 50 µm. M) Measurement of the length of the lateral edge shown in (I) and (K). Data are presented as mean ± SD (n = 10). Statistical analysis is performed using Student's *t*‐test (^****^ *P* < 0.0001). N) Measurement of the epidermal cell area shown in (J) and (L). Data are presented as mean ± SD (n = 237 cells for *oszhd1 oszhd2*, n = 260 cells for *pROC1::OsZHD1* in *oszhd1 oszhd2*). Statistical analysis is performed using Student's *t*‐test (^****^ *P* < 0.0001).

So far, our analyses demonstrate that disrupting the growth patterns of epidermal cells affects leaf angle formation. To further test the contribution of the epidermis to leaf angle formation, we generated a transgenic rice plant expressing the full *OsZHD1* genomic sequence driven by the epidermal specific *ROC1* promoter,^[^ ^28^, ^29^, ^30^ ^]^ which restored *OsZHD1* expression in epidermal cells of the mutant. Introduction of this transgene largely rescued the reduced flag leaf angle phenotype of *oszhd1 oszhd2*, resulting in a looser plant stature compared to *oszhd1 oszhd2* (**Figure** **5**A,B,D,E,G). Additionally, grains of *pROC1::OsZHD1* in *oszhd1 oszhd2* plants exhibited a significant increase in length, reversing the shorter grain phenotype observed in *oszhd1 oszhd2* (Figure S7, Supporting Information). These results indicate that *OsZHD1* can modify the morphology of both the flag leaf lamina joint and grains solely through restoring its activity in the epidermis.

Besides the flag leaf angle, the angle of the second complete leaf from 4‐week‐old seedlings of *pROC1::OsZHD1* in *oszhd1 oszhd2* was also restored (Figure 5C,F,H; Figure S8, Supporting Information). Observations of the mature lamina joint morphology under a stereoscope revealed that the length of the lamina joint on the lateral edge in *pROC1::OsZHD1* in *oszhd1 oszhd2* was significantly greater compared to *oszhd1 oszhd2* (Figure 5I,K,M). Given that *OsZHD1* acts as a positive regulator of leaf angle by promoting the proliferation and elongation of lateral epidermal cells at the lamina joint, we speculate that the number and area of epidermal cells on the lateral edge of the lamina joint in *pROC1::OsZHD1* in *oszhd1 oszhd2* were both restored, leading to an increase in the total length of the lateral edge of the lamina joint and consequently forming a larger leaf angle.

To test this hypothesis, we used Propidium Iodide (PI) staining to visualize epidermal cells in the mature lamina joint of the second complete leaf. We found that the area of epidermal cells on the lateral edge of the lamina joints of *pROC1::OsZHD1* in *oszhd1 oszhd2* was significantly increased compared to *oszhd1 oszhd2* (Figure 5J,L,N). However, due to PI's inability to stain the entire lamina joint, we could not accurately determine the number of epidermal cells along the lateral edge. Nonetheless, given that the total length of the lateral edge in *pROC1::OsZHD1* in *oszhd1 oszhd2* is ≈2.14 times that of *oszhd1 oszhd2*, and that the average area of epidermal cells is roughly 1.22 times greater, we infer that the number of epidermal cells in *pROC1::OsZHD1* in *oszhd1 oszhd2* has also increased to some extent.

### 2.6 OsZHD1 and OsZHD2 Regulate Leaf Angle Formation by Modulating Auxin Activity, as well as BR Signaling

To illuminate the underlying molecular mechanism of OsZHD1 and OsZHD2 in the regulation of lamina joint epidermal cell growth pattern and leaf angle, we generated transcriptome data of mature lamina joints from the second complete leaves for both WT and *oszhd1 oszhd2*. The reliability of our transcriptome data was validated through examining the expression levels of several significantly upregulated and downregulated genes, as well as key regulatory genes of leaf angle formation, in both WT and *oszhd1 oszhd2* (Figure S9, Supporting Information). A total of 563 differentially expressed genes (DEGs) were identified in *oszhd1 oszhd2* compared to WT, including 299 upregulated genes and 264 downregulated genes (Figure S10A and Table S3, Supporting Information). Kyoto Encyclopedia of Genes and Genome (KEGG) pathway analysis categorized the DEGs into diterpenoid biosynthesis, phenylpropanoid biosynthesis, cytochrome P450, limonene degradation, and tryptophan metabolism (Figure S10B, Supporting Information). Among these, the tryptophan metabolism pathway is of particular importance due to its role in the biosynthesis of auxin, a critical plant hormone that regulates leaf angle.^[^ ^31^ ^]^ The KEGG enrichment analysis results suggest that *OsZHD1* and *OsZHD2* may influence leaf angle by participating in the auxin pathway.

**Figure 6:**
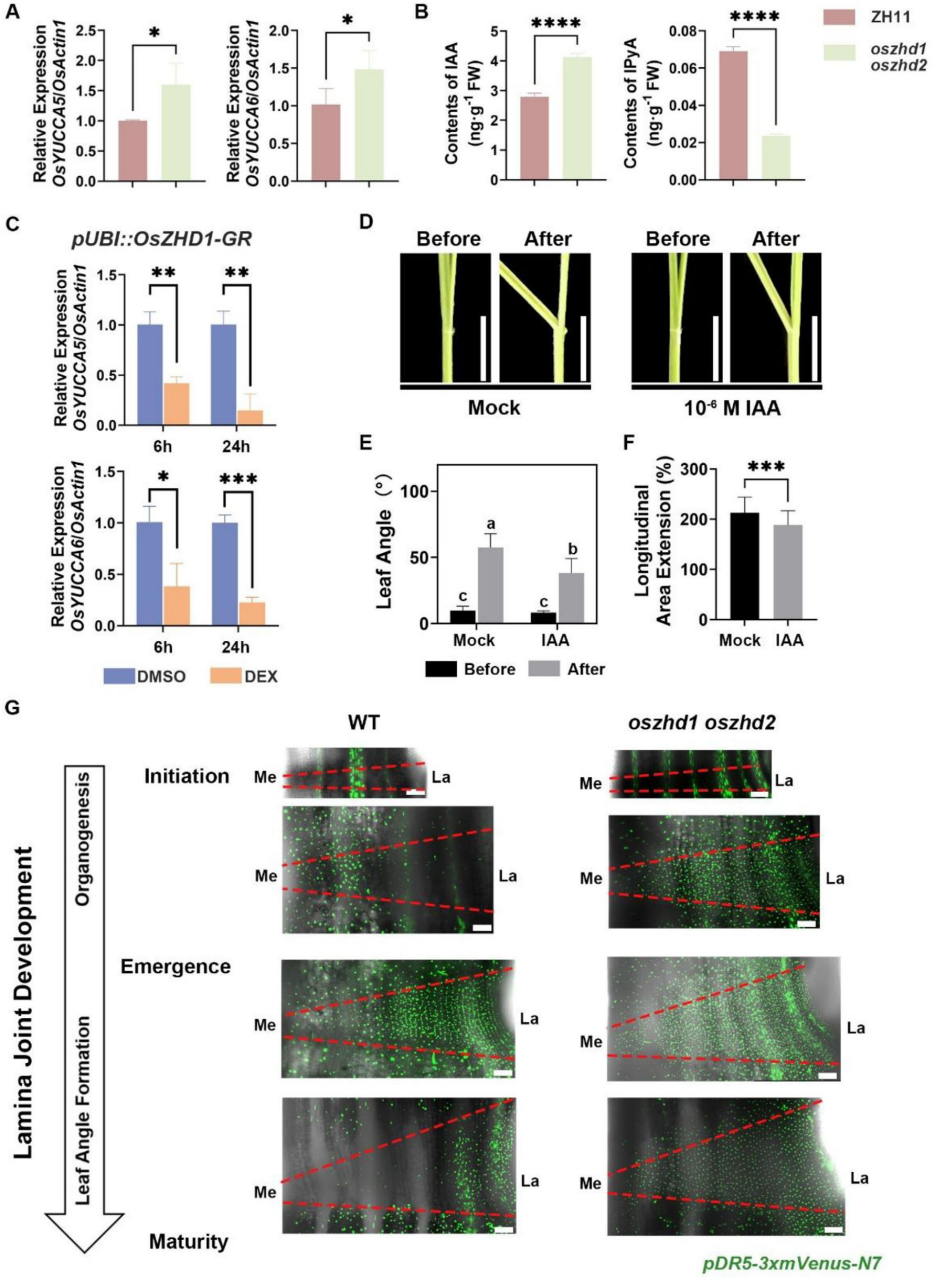
OsZHD1 and OsZHD2 regulate leaf angle formation by modulating auxin activity. A) Transcript levels of *OsYUCCA5* and *OsYUCCA6* analyzed by qRT‐PCR. *OsActin1* served as an internal control for normalization. Data are presented as mean ± SD (n = 3). Statistical analysis is performed using Student's *t*‐test (^*^ *P* < 0.05). B) Levels of IAA and IPyA in the mature lamina joints of the second complete leaves from 4‐week‐old seedlings. Data are presented as mean ± SD (n = 3). Statistical analysis is performed using Student's *t*‐test (^****^ *P* < 0.0001). C) Expression levels of *OsYUCCA5* and *OsYUCCA6* in *pUBI::OsZHD1‐GR* after DMSO (mock) or DEX treatment as determined by qRT‐PCR. Data are presented as means ± SD (n = 3). Statistical analysis is performed using Student's *t*‐test (^*^ *P* < 0.05, ^**^ *P* < 0.01, ^***^ *P* < 0.001). D) Lamina joint bending response to 10^−6^ m IAA. Scale bars, 0.5 cm. E) Quantification of the lamina joint bending assay described in (F). Data are presented as mean ± SD (n = 5). Statistical analysis is conducted using one‐way ANOVA followed by Tukey's post‐hoc test. Different letters indicate significant differences (*P* < 0.05). F) Quantification of longitudinal area extension (%) of epidermal cells. Data are presented as mean ± SD (n = 41 cells in the mock group, n = 50 cells in the IAA treatment group). Statistical analysis is performed using Student's *t*‐test (^***^ *P* < 0.001). G) Spatial distribution of the auxin response reporter *pDR5::3×mVenus‐N7* (DR5) during lamina joint development. Scale bars, 50 µm. n = 5 lamina joints.

In tryptophan‐dependent pathway for auxin biosynthesis, the TRYPTOPHAN AMINOTRANSFERASE OF ARABIDOPSIS (TAA) family of tryptophan aminotransferases first converts tryptophan to indole‐3‐pyruvate (IPyA), while the YUCCA family of flavin monooxygenases catalyzes the conversion of IPyA to indole‐3‐acetic acid (IAA).^[^ ^32^, ^33^, ^34^ ^]^ Accumulation of auxin negatively regulates leaf angle opening.^[^ ^12^, ^35^ ^]^ We found that the expression levels of *OsYUCCA5* and *OsYUCCA6*, two genes encoding enzymes for the rate‐limiting step in auxin biosynthesis, were significantly upregulated in RNA‐seq data (Figure S10C, Supporting Information). qRT‐PCR further confirmed the differential expression of these two genes between WT and *oszhd1 oszhd2* (**Figure** **6**A). To further confirm that the auxin content was altered in *oszhd1 oszhd2*, we measured the contents of IAA and auxin precursors in the lamina joints of the second complete leaves of both WT and *oszhd1 oszhd2*. The results showed that IAA content was significantly higher in *oszhd1 oszhd2* than in WT, while IPyA content was decreased (Figure 6B). Supporting a functional role for *OsYUCCA5* and *OsYUCCA6*, overexpression lines of both genes exhibited reduced leaf angles compared to WT (Figure S11, Supporting Information), consistent with the phenotype observed in *oszhd1 oszhd2*. In summary, we speculate that in *oszhd1 oszhd2*, the upregulation of *OsYUCCA5* and *OsYUCCA6* promotes the conversion of IPyA to IAA. Consequently, the IPyA content decreases while the IAA content increases in the *oszhd1 oszhd2*.

To further verify that *OsYUCCA5* and *OsYUCCA6* are regulated downstream by *OsZHD1* and *OsZHD2*, we generated transgenic plants in which a constitutively expressed Maize Ubiquitin promoter drove the expression of a fusion gene between *OsZHD1* and the rat glucocorticoid receptor (GR) coding region (*pUBI::OsZHD1‐GR*). Under dexamethasone (DEX) induction, *pUBI::OsZHD1‐GR* rapidly overexpresses *OsZHD1* in the nucleus, inducing transient changes in the expression levels of downstream genes. The mature lamina joints of 4‐week‐old *pUBI::OsZHD1‐GR* seedlings were harvested after 6 and 24 hours of DEX or DMSO treatment, and used for transcription analysis. Compared to the mock treatment, the expression levels of *OsYUCCA5* and *OsYUCCA6* were significantly downregulated after both 6 and 24 hours of DEX treatment, with levels decreasing further over time (Figure 6C). These results indicate that *OsYUCCA5* and *OsYUCCA6* are located downstream of OsZHD1. Based on our speculation that OsZHD1 and OsZHD2 have redundant functions in regulating leaf angle, we conclude that these two OsZHDs regulate the expression levels of *OsYUCCA5* and *OsYUCCA6* to mediate the synthesis of auxin in the lamina joint, thereby influencing the leaf angle phenotype.

We next tested whether auxin was sufficient to disrupt the patterns of the lamina joint epidermal development. The lamina joint of the second complete leaf from WT treated with 1 µM IAA exhibited a reduced leaf angle compared to the mock treatment (Figure 6D,E; Figure S12, Supporting Information). Following auxin treatment, the growth rate of the lamina joint epidermal cells decreased, demonstrating the negative regulatory effect of auxin on epidermal cell elongation and leaf angle formation (Figure 6F).

To further investigate the impact of *OsZHD1* and *OsZHD2* on auxin in lamina joint morphogenesis, we examined auxin activity patterns in WT and *oszhd1 oszhd2* lamina joints throughout development using the auxin response reporter *pDR5::3×mVenus‐N7* (DR5) (Figure 6G). In wild‐type lamina joints, DR5 signal distribution exhibited clear stage‐specific dynamics. No DR5 signal was detected in epidermal cells at the initial stage of lamina joint development. The signal subsequently appeared at the medial edge, expanded throughout the epidermis during lamina joint emergence, and later became enriched at the lateral edge during leaf angle formation. This asymmetric DR5 distribution closely paralleled the previously observed pattern of asymmetric epidermal cell elongation (Figure 2). In contrast, *oszhd1 oshzd2* lamina joints displayed disrupted DR5 signal dynamics: although no DR5 signal was present in epidermal cells during the initial stage, subsequently DR5 signals were distributed across the entire epidermis through the rest of the lamina joint developmental stages, lacking the asymmetric distribution pattern characteristic of WT lamina joints (Figure 6G). The increased distribution of DR5 signals in *oszhd1 oshzd2* lamina joints was consistent with the results shown by the transcriptomic analysis and metabolic assay mentioned above that *oszhd1 oshzd2* lamina joints had increased auxin levels. To further assess whether altered auxin activity affects leaf angle formation, we treated lamina joints with N‐1‐naphthylphthalamic acid (NPA), an inhibitor of directional auxin transport. NPA treatment significantly suppressed leaf angle formation, supporting that proper spatial distribution of auxin activity is essential for leaf angle regulation (Figure S13, Supporting Information). Together, these findings suggest that OsZHD1 and OsZHD2 regulate leaf angle formation, at least in part, by modulating auxin levels and maintaining appropriate spatial patterns of auxin activity within the lamina joint.

**Figure 7:**
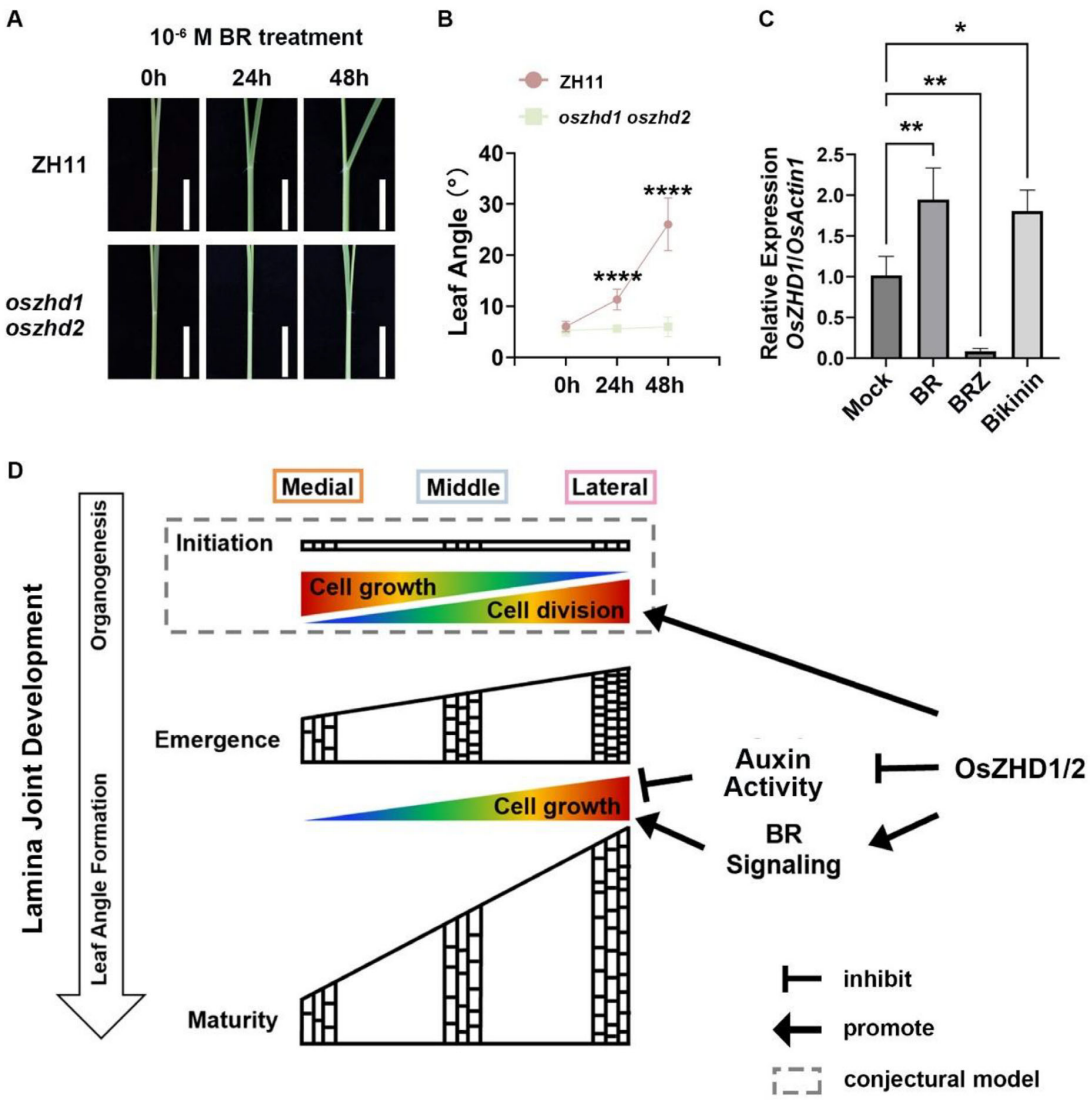
OsZHD1 and OsZHD2 contribute to leaf angle formation downstream of BR signaling. A) Lamina joint bending response to 10^−6^ M BR. Scale bars, 1 cm. B) Quantification of the lamina joint bending assay described in (A). Data are presented as mean ± SD (n  =  6). Statistical significance is determined using Student's *t*‐test (^****^ *P* < 0.0001). C) The relative changes of *OsZHD1* transcription levels in response to BR, BRZ, and bikinin. Data are presented as mean ± SD (n  =  3). Statistical analysis is conducted using one‐way ANOVA followed by Dunnett's post‐hoc test (^*^ *P* < 0.05, ^**^ *P* < 0.01). D) Schematic representations showing the impact of OsZHD1 and OsZHD2 on epidermal cell behavior in the rice lamina joint. This model summarizes the growth pattern of epidermal cells in the rice lamina joint and how OsZHD1/2 regulates this growth. During leaf angle formation, the epidermal cells of the lamina joint cease division and primarily elongate along the direction of the cell file. Spatial differences in cell number and elongation result in asymmetric growth between the lateral and medial edges, which contributes to leaf angle formation. Our data suggest that epidermal cell division is restricted to the organogenesis phase, during which there are spatial differences in both cell division and elongation in the lamina joint. OsZHD1/2 promote epidermal cell division during organogenesis and facilitate elongation by modulating auxin activity and BR signaling during leaf angle formation, thereby influencing lamina joint morphology and regulating leaf angle. The gray dashed box in the figure indicates a conjectural model that has not yet been directly verified in this study.

However, the limited inhibitory effect of auxin on leaf angle suggests that *OsZHD1* and *OsZHD2* may also regulate leaf angle through other physiological processes besides inhibiting auxin biosynthesis. Previous studies have shown that BRs profoundly affect the development of the lamina joint.^[^ ^11^, ^14^, ^36^ ^]^ To investigate whether BR signaling is also involved in *OsZHDs*’s regulation of leaf angle, we examined the leaf bending response of the lamina joint in WT and *oszhd1 oszhd2* following BR treatment. Compared to WT, *oszhd1 oszhd2* appeared largely insensitive to exogenous BRs (**Figure** **7**A,B; Figure S14, Supporting Information), suggesting that *OsZHDs* also play a role in BR's promotion of leaf angle formation. Furthermore, *OsZHD1* transcription levels increased significantly upon treatment with BR or Bikinin, a GSK3‐like kinase inhibitor that activates BR signaling, but decreased when treated with the BR biosynthesis inhibitor brassinazole (BRZ) (Figure 7C), indicating the impact of BRs on the expression of *OsZHD1*. Additionally, RNA‐seq analysis revealed no significant changes in the expression of early BR signaling genes (e.g., *OsBAK1*, *OsBRI1*, *OsBZR1*) in *oszhd1 oszhd2* (Figure S15A, Supporting Information). Similarly, marker genes acting early in the BR signaling pathway, such as *OsBZR1* ^[^ ^37^ ^]^ and *OsFLP*,^[^ ^11^ ^]^ showed the same expression changes in *oszhd1 oszhd2* as in WT—*OsBZR1* upregulated and *OsFLP* downregulated rapidly after BR treatment (Figure S15B,C, Supporting Information). In contrast, downstream targets of *OsBZR1*, such as *OsALDH2B1* ^[^ ^38^ ^]^ and *CYC U4;1*,^[^ ^14^ ^]^ were significantly mis‐regulated in *oszhd1 oszhd2* (Figure S15D, Supporting Information). Collectively, these findings suggest that *OsZHD1* and *OsZHD2* likely act downstream of BR signaling to modulate leaf angle formation.

All in all, our analyses fit with a model that OsZHD1 and OsZHD2 promote the division of lamina joint epidermal cells during organogenesis and facilitate their elongation by participating in auxin activity and BR signaling during leaf angle formation, thereby influencing lamina joint morphology and regulating leaf angle (Figure 7D).

## 3 Discussion

### 3.1 The Growth of Rice Lamina Joint Epidermal Cells Determines the Size of the Leaf Angle

The lamina joint serves as an ideal model for studying the regulatory mechanisms of cytology and organ development.^[^ ^8^ ^]^ In the study of rice lamina joint development, anatomical dissection and X‐ray microcomputed tomography are commonly employed to observe the cellular morphology of internal tissues and their contribution to lamina joint morphogenesis.^[^ ^10^, ^11^, ^12^, ^36^ ^]^ Despite the critical role of the epidermis in shaping organ morphology, its function and growth dynamics have often been overlooked in studies of lamina joint development. To address this gap, we generated a plasma membrane reporter (*pUBI::mCitrine‐RCI2A*) to dynamically track the behaviors of lamina joint epidermal cells during leaf angle formation. Our results demonstrate that the longitudinal elongation of lateral epidermal cells contributes to leaf angle formation. Furthermore, the mature leaf angle is positively correlated with the length of the lamina joint along the lateral edge, which is determined by both epidermal cell number and cell area (Figures 1 and 2). Cytological analysis reveals that *OsZHD1* and *OsZHD2* play key roles in regulating leaf angle by promoting both cell division and elongation of epidermal cells at the lateral edge of the lamina joint (Figures 3 and 4). Notably, restoring *OsZHD1* expression in the epidermis rescues the defective leaf angle phenotype of *oszhd1 oszhd2*. This restoration is accompanied by an increase in both the number of epidermal cells and their cell area compared to *oszhd1 oszhd2*, further emphasizing the significant impact of epidermal cell development on leaf angle formation (Figure 5).

Our study further supports the “epidermal growth control” theory, which proposes that epidermal cells can either drive or restrict the expansion of internal tissues, thereby regulating overall organ growth.^[^ ^20^, ^22^, ^39^ ^]^ Although we demonstrate for the first time the importance of epidermal cell development in lamina joint morphogenesis, the influence of lamina joint internal tissues on the overall organ morphology cannot be overlooked. Previous studies have shown that lamina joint development involves multiple cellular processes within internal tissue and have suggested that leaf angle is determined by the balance between the pushing force generated by expanding parenchymal cells and the supporting force provided by sclerenchyma cells.^[^ ^8^, ^10^ ^]^ Our dynamic observations of epidermal cells in the lamina joint are consistent with the previously reported behavior of internal cells. For instance, cell division occurs in both epidermal and internal cells before stage 4. During leaf angle formation, from stage 4 to stage 6 of lamina joint development, both epidermal and parenchyma cells undergo cell elongation (Figure 2; Figure S3, Supporting Information). These results underscore the significant role of epidermal cell development in regulating lamina joint morphology alongside internal tissues.

Plant cells are interconnected by their cell walls, and the coordinated growth of different cell layers and tissue types is essential for the formation of physiologically efficient organs.^[^ ^20^, ^24^, ^40^, ^41^ ^]^ Therefore, the proper establishment of organ morphology is closely tied to the coordinated growth of epidermal and internal tissues. Recent studies have further reported that organ morphogenesis depends on mechanical interactions between cells and tissues, where these interactions generate forces that are sensed by cells and influence key cellular processes.^[^ ^40^ ^]^ While our results highlight the coordinated growth of epidermal and internal tissues, how mechanical forces and signaling networks jointly contribute to lamina joint morphogenesis and leaf angle formation remains largely unclear. Due to the suitability of the lamina joint for dynamic growth tracking of both epidermal and internal tissues, it serves as an ideal organ in rice to observe the coordination of cell behaviors during morphogenesis. Further research will focus on exploring the coordination and underlying mechanisms between epidermis and internal tissues during lamina joint morphogenesis.

### 3.2 Spatial and Temporal Control of Epidermal Cell Growth Directs the Rice Lamina Joint Morphology

Since dynamic changes in cells determine the morphology of organs, understanding organ morphogenesis requires precisely linking the spatiotemporal behavior of cells with changes in organ morphology, which is currently a hot and challenging topic in developmental biology research.^[^ ^42^, ^43^ ^]^ In model plants such as *Arabidopsis* and *Antirrhinum*, real‐time live imaging of organ development has greatly advanced the study of the mechanisms controlling morphogenesis.^[^ ^44^, ^45^ ^]^ Monocotyledonous plants have distinct morphological and anatomical structures compared to dicotyledonous plants. How monocots regulate cell growth, division, and differentiation during development to maintain specific organ morphology remains to be elucidated. To address these unresolved issues, our study specifically focused on the cellular dynamics of the rice lamina joint, developing a non‐destructive method by using CLSM to investigate the detailed behavior of epidermal cells during leaf angle formation. Our research identified the lamina joint as an ideal organ for tracking cell behaviors in morphogenesis in rice for several reasons: (1) compared to leaves and sheaths, the epidermal cells of the lamina joint have less wax and papillae, resulting in reduced interference from plant tissue autofluorescence when using fluorescent protein reporters; (2) during most of the period in which the lamina joint undergoes morphological changes, it remains exposed rather than enclosed by other organs, making it convenient for microscopic observation and manipulation; and (3) the lamina joint is relatively small, allowing the growth of the entire organ to be observed under a high‐power microscope.

In plants, the precise spatial and temporal control of cell proliferation and expansion determines the differential growth that defines organ shape and size.^[^ ^46^ ^]^ Our results demonstrate that during leaf angle formation, the lamina joint epidermal cells cease dividing (Figure 2; Figure S3, Supporting Information). Previously, transcriptome analysis revealed that genes involved in stage 1 to stage 3 are primarily associated with cell division, which aligns with the cytological features observed in the lamina joint inner cells.^[^ ^8^, ^10^ ^]^ We deduce that cell division in the epidermal cells of the lamina joint is primarily restricted to the early stages of development, indicating the stage‐specific regulation of cell division. The differences in cell numbers between the lateral and medial edges in the mature lamina joint suggest varying levels of early cell division activity at different regions of the lamina joint at the organogenesis phase. Specifically, cells near the lateral edge undergo more division, while those near the medial edge divide less (Figure 7).

In addition to the spatial differences in cell division during lamina joint organogenesis, spatial differences in cell elongation during leaf angle formation are key factors of leaf angle size. During this phase, cells near the lateral edge elongate faster, while cells near the medial edge almost cease growing (Figures 2 and 7). A similar spatial pattern is observed in both inner and epidermal cells during leaf angle formation. Previous studies have shown spatial differences in lamina joint inner cell elongation: during stage 5, the abaxial parenchyma cells stop growing, while the remaining parenchyma cells on the adaxial edge continue to undergo vertical elongation.^[^ ^8^ ^]^

Although we were unable to image the epidermal cells of the lamina joint during organogenesis due to anatomical restriction and imaging limitations, we observed that by the emergence stage, the epidermal cells on the lateral edge were significantly smaller than those on the medial edge, despite the larger number of cells on the lateral edge (Figures 1 and 2). We deduce that there are also spatial variations in the elongation of lamina joint epidermal cells during organogenesis. Specifically, we propose that the epidermal cells on the medial edge compensate for reduced cell division by promoting greater cell elongation, leading to a relatively equal length between the medial and lateral edges. This balanced edge length may facilitate better wrapping by the leaf sheath during organogenesis of the lamina joint (Figure 7).

### 3.3 OsZHD1 and OsZHD2 Regulate Leaf Angle through Multiple Hormone Pathways

Auxin has been reported to negatively regulate the leaf angle development.^[^ ^19^, ^47^, ^48^ ^]^ We found that *OsZHD1* and *OsZHD2* negatively regulate the expression of the rate‐limiting enzymes of auxin synthesis *OsYUCCA5* and *OsYUCCA6*, thereby reducing auxin accumulation in the lamina joint. Since reduced auxin levels lead to enlarged leaf angles by promoting parenchyma cell division and elongation at the adaxial side,^[^ ^17^, ^18^, ^19^ ^]^ we propose that the increased IAA levels in *oszhd1 oszhd2* might inhibit the cell growth in the epidermal cells.

The regulatory effects of *OsZHD1* and *OsZHD2* on auxin accumulation may vary across different organs. *OsZHD2* can promote ethylene synthesis by positively regulating *OsACS5*, which in turn induces auxin accumulation in roots and affects the activity of rice root meristematic tissues.^[^ ^26^ ^]^ During inflorescence meristem development, *OsZHD1* and *OsZHD2* also promote flowering by positively regulating auxin synthesis.^[^ ^27^ ^]^ Previous studies have reported that *OsZHD1* and *OsZHD2* induce auxin accumulation by positively regulating *OsYUCCA7* in the root and inflorescence meristem, which seems to contrast with our findings that *OsZHD1* and *OsZHD2* reduce auxin accumulation by negatively regulating the expression of *OsYUCCA5* and *OsYUCCA6* in the lamina joint. To further investigate the tissue‐specific regulation of *OsZHD1* and *OsZHD2* on YUCCA genes, we examined YUCCA gene expression in different tissues (Figure S16, Supporting Information). We extracted RNA from the roots of ZH11 and *oszhd1 oszhd2* and used *OsACS5*, a direct downstream target of OsZHD2, as a reference. In roots, we found no significant difference in *OsYUCCA5* expression between ZH11 and *oszhd1 oszhd2*, while *OsYUCCA6* was significantly upregulated in *oszhd1 oszhd2*. We also measured *OsYUCCA5* and *OsYUCCA6* expression at both the emergence and maturity stages of lamina joint development, and found consistently higher levels in *oszhd1 oszhd2* compared to ZH11. These results suggest that OsZHD1 and OsZHD2 may regulate YUCCA genes in a tissue type dependent manner.

Furthermore, we investigated the transcriptional regulatory function of OsZHD1 using both tobacco and rice protoplast systems (Figure S17, Supporting Information). Our results indicate that OsZHD1 functions as a transcriptional repressor. Given that transcriptional regulation by ZF‐HD family members often depends on interacting proteins,^[^ ^49^ ^]^ and that some transcription factors exhibit different transcriptional regulation on their target genes,^[^ ^38^ ^]^ we propose that OsZHD1 may negatively regulate *OsYUCCA5* and *OsYUCCA6* in the lamina joint. Notably, previous studies have not identified any member of the YUCCA family as direct downstream targets of *OsZHD1* or *OsZHD2*. Therefore, we propose that *OsZHD1* and *OsZHD2* may exert different transcriptional regulatory effects on various direct downstream genes through interactions with different proteins, thereby influencing the expression of the YUCCA family. Considering that *OsYUCCA5* and *OsYUCCA6* can be rapidly induced by DEX in *pUBI::OsZHD1‐GR* lines, we speculate that *OsYUCCA5* and *OsYUCCA6* may be potential targets of OsZHD1. The relationship of OsYUCCA5 and OsYUCCA6 with OsZHD1 in the modulation of leaf angle requires further investigation.

In addition to the previously reported involvement of OsZHD1 and OsZHD2 in the ethylene and auxin pathways, our study reveals that OsZHD1 and OsZHD2 also participate in the BR signaling pathway as transcription factors regulating epidermal development. BR application experiments suggest that OsZHD1 and OsZHD2 act downstream of BR signaling to modulate lamina joint development and leaf angle formation (Figure 7A,B; Figures S14 and S15, Supporting Information). Previous genetic and mechanical studies have demonstrated that BR signaling in epidermal cells is crucial for promoting organ growth and coordinating mechanical interactions between tissue layers.^[^ ^22^, ^24^, ^50^, ^51^ ^]^ Given that BR is a key hormonal regulator of lamina joint development,^[^ ^11^, ^14^, ^36^ ^]^ and that the epidermis serves as the primary site of BR action,^[^ ^22^, ^24^ ^]^ our findings further support the conclusion that epidermal cell dynamics is a crucial determinant of lamina joint morphogenesis.

Emerging evidence also highlights the coordinated regulation of cell growth between the adaxial and abaxial sides of the lamina joint through the interaction of BR and auxin at different developmental stages.^[^ ^9^ ^]^ Consistent with this, our results indicate that OsZHD1 and OsZHD2 function as key transcription factors involved in BR‐auxin pathways, echoing previous reports that most auxin‐related mutants present changed BR sensitivity.^[^ ^19^, ^48^ ^]^ Collectively, these findings suggest that OsZHD1 and OsZHD2 regulate organ development by participating in multiple hormone pathways including auxin, BR, and ethylene, and likely serve as molecular links connecting hormonal signaling with epidermal mechanical regulation that shapes organ morphology.

Given that OsZHD1 and OsZHD2 function as key transcription factors integrating multiple hormonal pathways to regulate epidermal cell dynamics and lamina joint development, we further examined whether their regulatory roles are evolutionarily conserved. A total of 103 ZF‐HD proteins were identified across five representative monocot species, including 15 from rice, 24 from maize, 12 from barley, 15 from foxtail millet, and 37 from wheat (Figure S18, Supporting Information). Phylogenetic analysis revealed nine closest orthologs of OsZHD1 and OsZHD2 (Figure S18, Supporting Information), and sequence alignment showed strong conservation, particularly within the zinc‐finger and homeodomain regions in these orthologs (Figure S19A,B, Supporting Information). Single‐cell RNA‐seq^[^ ^52^ ^]^ indicated that OsZHD1 and OsZHD2 are expressed in both epidermal and inner tissues (Figure S19C, Supporting Information). Notably, epidermis‐specific restoration of *OsZHD1* expression rescued the lamina joint defect of the *oszhd1 oszhd2*, underscoring the critical role of epidermal regulation in leaf angle formation. Together with the conserved amino acid sequence of orthologs and previous evidence that *ZmZHD1* and *ZmZHD21* regulate leaf angle in maize,^[^ ^53^ ^]^ these findings suggest that the ZF‐HD mediated regulation of leaf angle—likely through the control of epidermal cell dynamics—probably represents a highly conserved developmental mechanism underlying leaf architecture across the grass family.

## 4 Experimental Section

### Plant Materials and Growth Conditions

*Oryza sativa* L. ssp. *japonica* cv. ZH11 was used as the WT control for mutants throughout. *oszhd1 oszhd2* were generated using CRISPR‐Cas9 technology. *oszhd1* and *oszhd2* were isolated from the progeny of an *oszhd1 oszhd2* x ZH11 cross. Multiple strains with various editing types were obtained, which are listed in Table S1 (Supporting Information). Crossing *oszhd1 oszhd2* with *pUBI::mCitrine‐RCI2A* introduced the plasma membrane reporter into the *oszhd1 oszhd2* background.

Rice plants were cultivated under field conditions at experimental stations in Changxing (31°N, 119°E) during the summer for agronomic trait analyses, and in Lingshui (18°N, 110°E) during the winter for seed propagation. For convenience, non‐transgenic materials were occasionally grown in Hangzhou (30°N, 120°E) during the summer, which has a similar latitude to Changxing. However, all agronomic measurements were obtained from plants grown in Changxing.

For seedling phenotypic and lamina joint epidermal cell analyses, plants were cultivated in an incubator under controlled conditions. Seeds were soaked in distilled water at room temperature for 2 days, followed by 2 days of imbibition in water at 37 °C in a constant‐temperature incubator. The germinated seeds were transferred to 96‐well plates with cut bottoms and grown in water under a 16‐hour light (30 °C)/8‐hour dark (26 °C) cycle. Each plant was spaced at least 3 cm apart to allow sufficient room for the second complete leaf to fully unfold.

### Transgenic Plants

To generate *pUBI::mCitrine‐RCI2A*, the *mCitrine‐RCI2A* sequence was amplified from *pATML1::mCitrine‐RCI2A* ^[^ ^44^ ^]^ using KOD DNA polymerase (Toyobo, CAT KOD‐101) with primers oSX20 and oSX21. The entire sequence was then cloned into *pCUbi1390* (Maize ubiquitin promoter inserted into *pCAMBIA1390*) digested with *KpnI* and *PstI* using T5 exonuclease‐dependent assembly.^[^ ^54^ ^]^ Positive seedlings were identified using laser confocal microscopy, and strains showing strong signals were selected for subsequent experiments.

To knock out the *OsZHD1* and *OsZHD2* genes, the study designed target sequences with complementary NGG PAM structures from CRISPR‐P^[^ ^55^ ^]^ for both genes and then cloned them separately into *pEntry A* ^[^ ^56^ ^]^ and *pEntry B*,^[^ ^56^ ^]^ each digested with *BsaI*. T4 DNA Ligase (NEB, CAT M0202S) was used to insert two sgRNAs into *pRHCas9*,^[^ ^56^ ^]^ which had been digested with *PstI* and *SpeI*. Genotyping and sequencing of the transgenic plants were performed to confirm the edit type of *OsZHD1* (using primers oYX118 and oYX125) and *OsZHD2* (using primers oYX120 and oYX126).

The 2873 bp promoter of *ROC1* was first PCR‐amplified using primers oYX362 and oYX363 from WT genomic DNA, then cloned into *pRGE*,^[^ ^56^ ^]^ which had been digested with *PstI* and *BamHI*, to generate *pYX103*. The *OsZHD1* coding region was amplified using primers oYX386 and oYX387, and subsequently inserted into *pYX103*, digested with *SacI* and *SpeI*, to generate *pROC1::OsZHD1*.

For the construction of *pUBI::OsZHD1‐GR*, the *OsZHD1* coding region was amplified using primers oYX202 and oYX190. The GR segment was cloned from *p35S::ARF3m‐GR* ^[^ ^57^ ^]^ using primers oYX188 and oYX185. These two segments were fused into the *BamHI* and *SpeI* sites of the binary vector *pCUbi1390* using MultiF Seamless Assembly Mix (ABclonal, CAT RK21020).

To generate *pDR5::3×mVenus‐N7* transgenic rice lines in both ZH11 and *oszhd1 oszhd2* backgrounds, the plasmid was obtained from Wang Lab.^[^ ^58^ ^]^ This plasmid contains a synthetic auxin‐responsive promoter (*pDR5*) driving triple tandem repeats of nuclear‐localized mVenus fluorescent protein (*3×mVenus‐N7*), allowing sensitive visualization of auxin activity. Positive seedlings were identified by laser confocal microscopy.

For *pUBI::OsYUCCA5* and *pUBI::OsYUCCA6* constructs, the *OsYUCCA5* and *OsYUCCA6* coding sequences were amplified using primer pairs oYX535/oYX536 and oYX547/oYX548, respectively, with 2× Phanta Flash Master Mix (Vazyme, CAT P520). The PCR products were cloned into *pCUbi1390* digested with *KpnI* and *SpeI* using T5 exonuclease‐dependent assembly.^[^ ^54^ ^]^

All primers used for vector construction and genotyping are listed in Table S2 (Supporting Information). All final constructs were verified by sequencing and transformed into the corresponding plants through *Agrobacterium*‐mediated genetic transformation by Biorun Biosciences Company.

### The Developmental Stages of the Lamina Joint

The second complete leaf was selected for analysis. The developmental process of lamina joints was divided into six successive stages based on the morphological features of developing lamina joints.^[^ ^8^, ^11^ ^]^ The primary observations in this study focused on the emergence to maturity stages, specifically from the beginning of stage 4, when the lamina joint emerges from the previous leaf sheath and is exposed to sunlight, to the end of stage 6, when the leaf angle of the second complete leaf reaches its maximum.

### Scanning Electron Microscopy (SEM)

The samples were fixed in FAA solution (3.7% formaldehyde, 5% acetic acid, 50% ethanol), evacuated for one hour, and then preserved for more than 12 hours. The fixative was poured off, and the samples were rinsed three times with 0.1 m phosphate buffer solution (pH 7.0) for 15 minutes each time. The samples were then fixed with 1% osmium acid solution for 1–2 hours. The osmium acid waste was carefully removed, and the samples were rinsed three times with 0.1 m phosphate buffer solution (pH 7.0) for 15 minutes each time. The samples were dehydrated using an ethanol gradient (30%, 50%, 70%, 80%, 90%, and 95%), with each concentration applied for 15 minutes, followed by treatment with 100% ethanol for 20 minutes. Finally, the ethanol was replaced with fresh 100% ethanol. The samples were dried using a Hitachi HCP‐2 critical point dryer. The dehydrated samples were coated with gold‐palladium in a Hitachi Model E‐1010 ion sputter for 4–5 minutes and observed under a Hitachi Model SU‐8010 SEM.

### Live Imaging and Cell Behavior Analysis

To observe the development of lamina joint epidermal cells, *pUBI::mCitrine‐RCI2A* was used. Under the growth conditions, it takes about 2 weeks for the second complete leaf lamina joint to reach the emergence stage. At this point, the entire seedling was fixed sideways on a glass slide, and one side of the lamina joint was imaged. The imaged side of the lamina joint was marked to ensure consistent observation of the same side in future sessions. The roots of the imaged samples were kept moist during the imaging to prevent the rice seedlings from drying out. Imaging was conducted using a Nikon C2si confocal microscope with a 20X objective. The imaging settings were: excitation laser at 488 nm, emission collection range of 500–550 nm, and a z‐step of 0.5 µm. After imaging, the plants were returned to the growth chamber for subsequent imaging sessions. Images were processed using ImageJ (https://imagej.net/software/imagej/) to convert ND2 file to TIFF format. Growth analyses were performed with MorphographX, following previously described methods.^[^ ^59^ ^]^

### RNA‐Seq

Seedlings were sampled by excising ≈2 cm segments containing the mature second complete leaf lamina joint, part of leaf blade, and part of leaf sheath from both WT and *oszhd1 oszhd2*. Three biological replicates were collected, with each replicate combining lamina joints from different individual plants. Total RNA was extracted using Trizol (ABclonal, CAT RK30129) following the manufacturer's instructions. Library preparation was conducted, and their quality was assessed using an Agilent 2100 Bioanalyzer (Agilent Technologies). Sequencing was subsequently performed on a HiSeq 2500 (Illumina) according to the manufacturer's guidelines. Raw reads were cleaned and aligned to the ZH11 reference genome using HISAT2.^[^ ^60^ ^]^ Genes with more than a twofold change in expression and a *p*‐value ≤ 0.05 were considered to be differentially expressed genes.

### Quantitative Reverse Transcription Polymerase Chain Reaction (qRT‐PCR)

Total RNA was extracted as described above. After removing residual gDNA, cDNA was synthesized using the PrimeScript RT Reagent Kit with gDNA Eraser (Takara, CAT RR047A). qPCR was performed using the Hieff qPCR SYBR Green Master Mix (Yeasen, CAT 11204ES08) on a Bio‐Rad CFX96 Real‐Time PCR System (Bio‐Rad). Three biological replicates were conducted. *OsActin1* quantification was used as the control. All primers used for qRT‐PCR are listed in Table S2 (Supporting Information).

### DEX Treatment

*pUBI::OsZHD1‐GR* plants were grown in water‐filled 96‐well plates with cut bottoms, as described previously. It takes approximately 4 weeks for the second complete leaf lamina joint to reach stage 5. At this point, the water was replaced with either a 10 µm DEX solution or a 10 µm DMSO solution (as the mock treatment). Simultaneously, a 10 µm DEX or DMSO solution was sprayed onto the lamina joint. After 6 and 24 hours of DEX or DMSO treatment, segments of ≈2 cm, containing the second complete leaf lamina joint, leaf blade, and leaf sheath, were dissected and immediately frozen in liquid nitrogen for RNA extraction and qRT‐PCR analysis. The statistical analysis was conducted with Student's *t*‐test.

### Hormone Treatment

Seedlings were grown in water‐filled 96‐well plates with cut bottoms, as previously described.

For lamina joint in vitro culture with IAA, NPA, or BR, two‐week‐old seedlings were used by excising ≈2 cm segments containing the lamina joint, leaf blade, and leaf sheath of the second complete leaf. The excised segments were floated on sterile water for 10 min and then transferred to hormone solutions at the indicated concentrations. The lamina joints were incubated at 28 °C in the dark for 2 days, as described before.^[^ ^36^ ^]^

For lamina joint live cultivation with IAA or BR, the study followed the protocol of Huang et al.^[^ ^12^ ^]^ In the IAA live cultivation assay, two‐week‐old ZH11 seedlings were treated with 10^−6^ m IAA, and leaf angles were measured every 2 days over a period of 8 days. For BR live cultivation, two‐week‐old ZH11 and *oszhd1 oszhd2* seedlings were treated with 10^−6^ m BR, and leaf angles were measured daily for 2 days.

For gene expression analysis after hormone treatment, two‐week‐old seedlings were treated with 10^−6^ m BR, 10^−6^ m BRZ, or 10^−6^ m Bikinin. At the start of treatment, water was replaced with hormone or DMSO solution (mock control), and the same solution was simultaneously sprayed onto the lamina joint. After 4 h of treatment, ≈2 cm segments containing the lamina joint, leaf blade, and leaf sheath of the second complete leaf were excised and immediately frozen in liquid nitrogen for RNA extraction and qRT‐PCR analysis. The statistical analysis was conducted with Student's *t*‐test.

### PI Staining

Seedlings were sampled by excising ≈2 cm segments containing the mature second complete leaf lamina joint, along with parts of the leaf blade and sheath, while avoiding damage to the surface of the lamina joint. The sample was placed in a 100 µg mL^−1^ PI solution for staining at room temperature for 20–30 minutes. Insufficient staining time would result in incomplete staining, while prolonged staining could cause cell death as the dye penetrates the cell membrane and binds to the nucleus. After staining, the sample was rinsed twice with sterile water to prevent background noise from excess PI dye during imaging. The rinsed sample was then placed on a slide for imaging observation.

### Quantification of Phytohormones

Approximately 200 mg (fresh weight) of mature lamina joints, along with parts of the leaf blade and sheath, were collected from 4‐week‐old ZH11 and *oszhd1 oszhd2* plants for analysis (n ≥ 100, with 3 technical replicates). Phytohormones quantification was performed using a high performance liquid chromatography‐mass spectrometry (HPLC‐MS) system at Nanjing Ruiyuan Biotechnology Co., Ltd.

### Statistical Analysis

All statistical analyses were conducted using GraphPad Prism. Bar graph data are presented as mean ± SD. In box plots, the central line indicates the median, and the box boundaries represent the 25th and 75th percentiles. Outliers beyond the 5th and 95th percentiles are shown as individual dots. For comparisons between two groups, statistical significance was assessed using Student's *t*‐test. Differences among three or more groups were analyzed by one‐way ANOVA followed by Tukey's post hoc‐test for multiple comparisons, or Dunnett's post hoc‐test when each treatment was compared with the control. Significance levels are denoted as follows: ^*^ *P* < 0.05, ^**^ *P* < 0.01, ^***^ *P* < 0.001, and ^****^ *P* < 0.0001. Different letters indicate statistically significant differences (*P* < 0.05), whereas identical letters denote no significant difference (*P* > 0.05). Sample sizes for each statistical analysis are provided in the corresponding figure legends.

### Accession Numbers

Sequence from this study can be download from the rice annotation project database (https://rapdb.dna.affrc.go.jp/index.html) with the following accession numbers: *OsZHD1* (Os09g0466400); *OsZHD2* (Os08g0479400); *ROC1* (Os08g0187500); *OsYUCCA5* (Os12g0512000); *OsYUCCA6* (Os07g0437000); *OsACS5* (Os01g0192900); *OsALDH2B1* (Os06g0270900); *OsBGlu30* (Os09g0491100); *OsPR1aL* (Os07g0129300); *OsAP77* (Os10g0537800); *OsLG1* (Os04g0656500); *OsLG2* (Os01g0859500); *CYC U4;1* (Os10g0563900); *OsEXP1* (Os04g0228400); *OsEXP4* (Os05g0477600); *OsCESA1* (Os05g0176100); *OsCESA3* (Os07g0424400); *OsXTH9* (Os04g0604300); *OsGNS8* (Os05g0495900); *OsWAK60* (Os04g0370900); *OsBAK1* (Os08g0174700); *OsBRI1* (Os01g0718300); *OsBZR1* (Os07g0580500); *OsFLP* (Os07g0627300); *OsActin1* (Os03g0718100).

## Author Contributions

Y.X. and L.H. conceptualized the idea and designed the experiments. Y.X., H.Z., W.C., S.X., X.H., D.X., and X.R. performed experiments. Y.X., W.C., and L.H. performed live imaging and analysis. X.W. and M.Z. performed RNA‐seq analysis. Y.X. and L.H. performed manuscript writing. Y.X., H.Z., X.W., W.C., S.X., X.H., D.X., M.Z., and L.H. contributed to manuscript revising and editing.

## Conflict of Interest

The authors declare no conflict of interest.

## Supporting information



Supporting Information

Supporting Information

## Data Availability

The data that support the findings of this study are available in the supplementary material of this article.
