# Supplementary material for: Epidermal Cell Dynamics Regulates Rice Lamina Joint Morphogenesis and Leaf Angle Formation through OsZHD1 and OsZHD2 Regulation
Source: Adv Sci (Weinh). 2025 Dec 3;13(9):e18691. doi: 10.1002/advs.202518691 (PMC12903967; doi:10.1002/advs.202518691)
Supplement: Supplementary file 1 — Supporting Information [file ADVS-13-e18691-s001.docx]

**
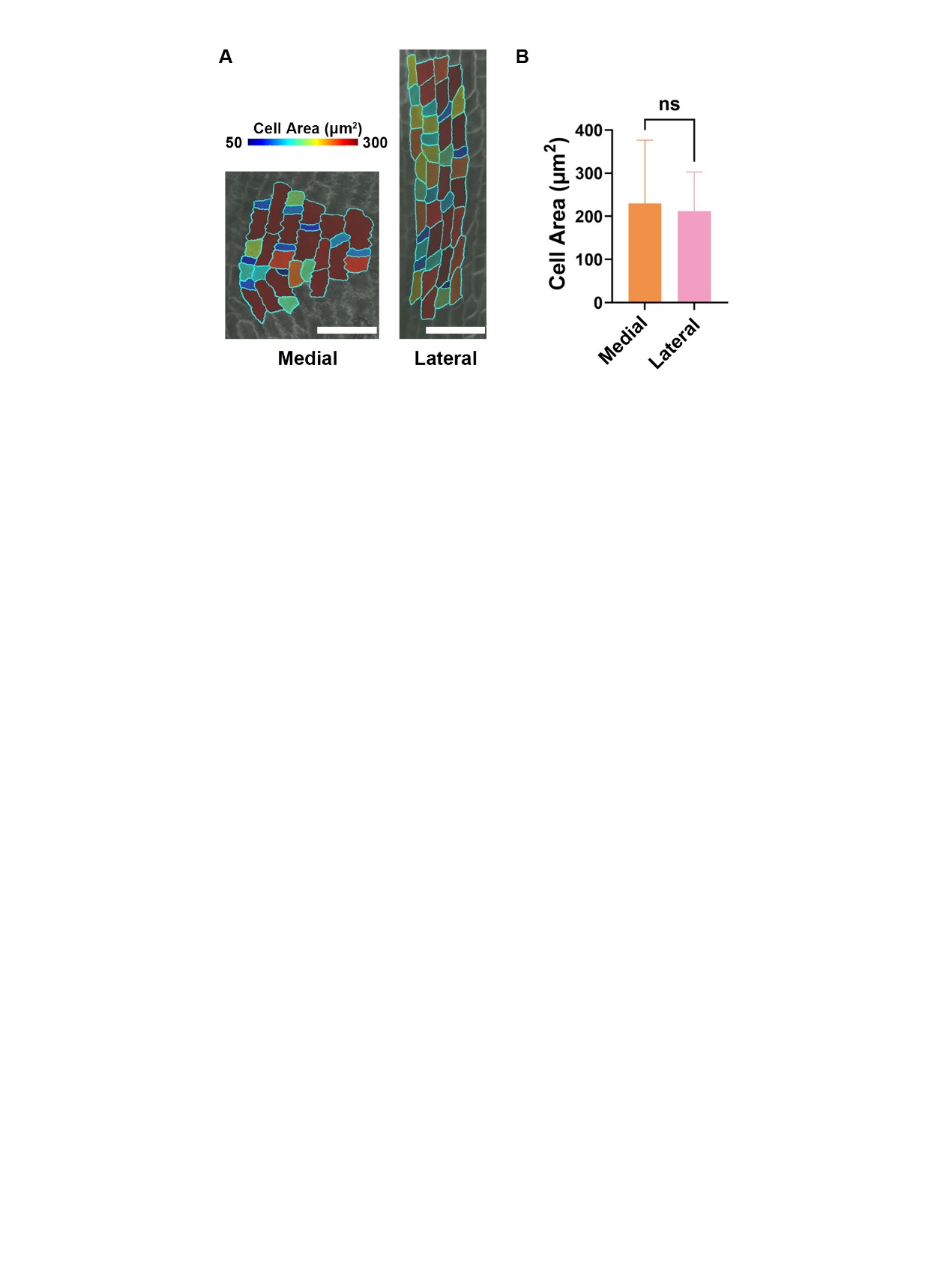
**

**Fig.S1 There is no significant difference in epidermal cell areas between the medial and lateral edges.**

**(A)** Heatmaps of cell areas in the medial and lateral edges. Scale bars, 50 µm.

**(B)** Cell area analysis. Data are presented as mean ± SD (n = 70 cells for the medial edge, n = 86 cells for the lateral edge, from 3 lamina joints). Statistical analysis is performed using Student’s t-test (ns = no significance).


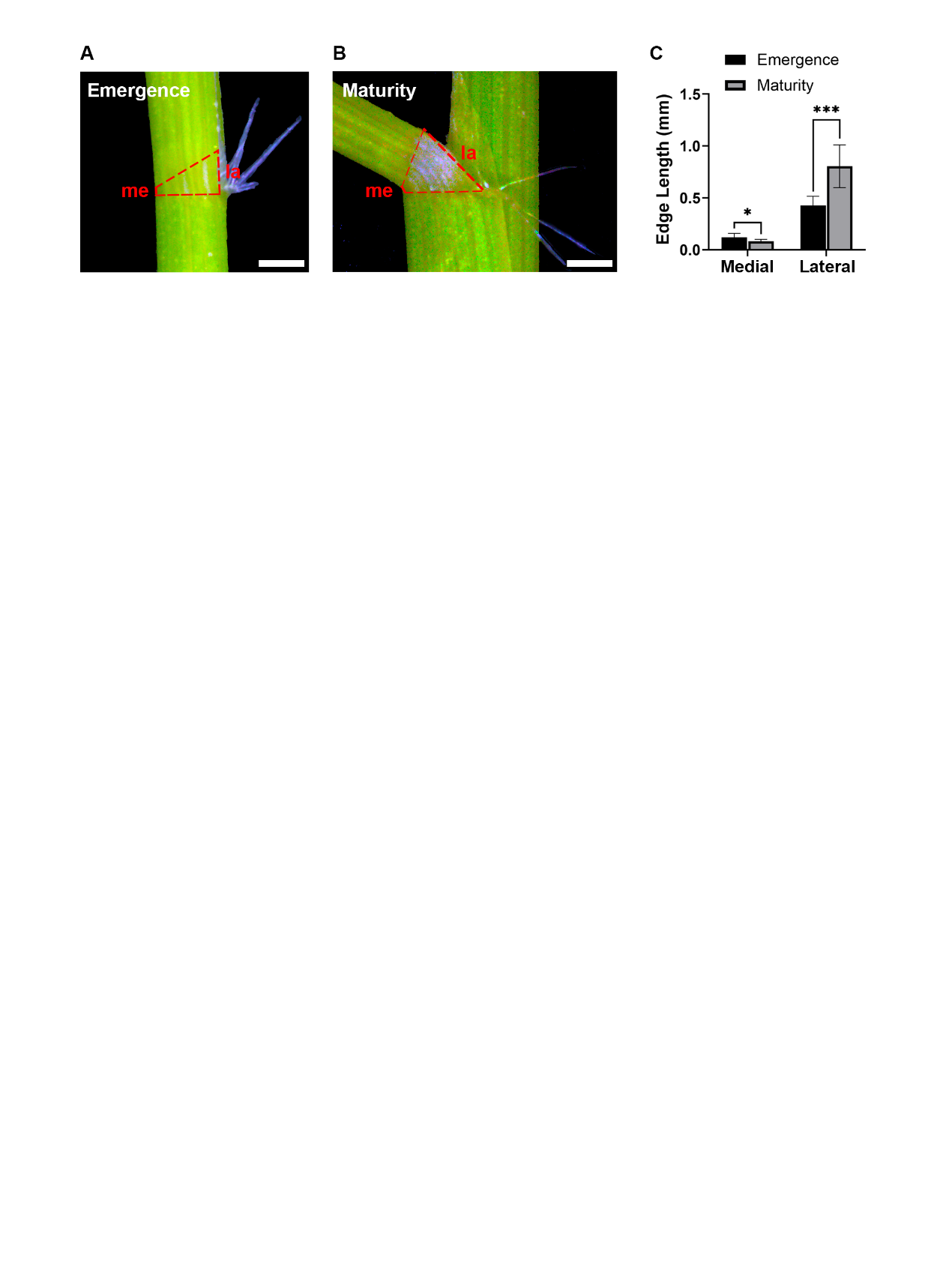


**Fig.S2 There is a significant increase in the length of the lateral edge during leaf angle formation.**

**(A)** The red dashed lines outlined the area of the lamina joint of the second complete leaf from a 15-day-old rice seedling. The lamina joint is at the emergence stage and begins to be exposed to sunlight (the first complete leaf was removed to facilitate observation of the lamina joint in the second complete leaf). Scale bar, 0.5 mm.

**(B)** The red dashed lines outlined the area of the lamina joint of the second complete leaf from a 27-day-old rice seedling. The lamina joint is at the maturity stage, and the leaf angle of the second complete leaf reaches its maximum. Scale bar, 0.5 mm.

**(C)** Edge length analysis of the lamina joint of the second complete leaf at different developmental stages. Data are presented as mean ± SD (n = 7). Statistical significance is determined using Student’s t-test (^*^P < 0.05, ^***^P < 0.001).


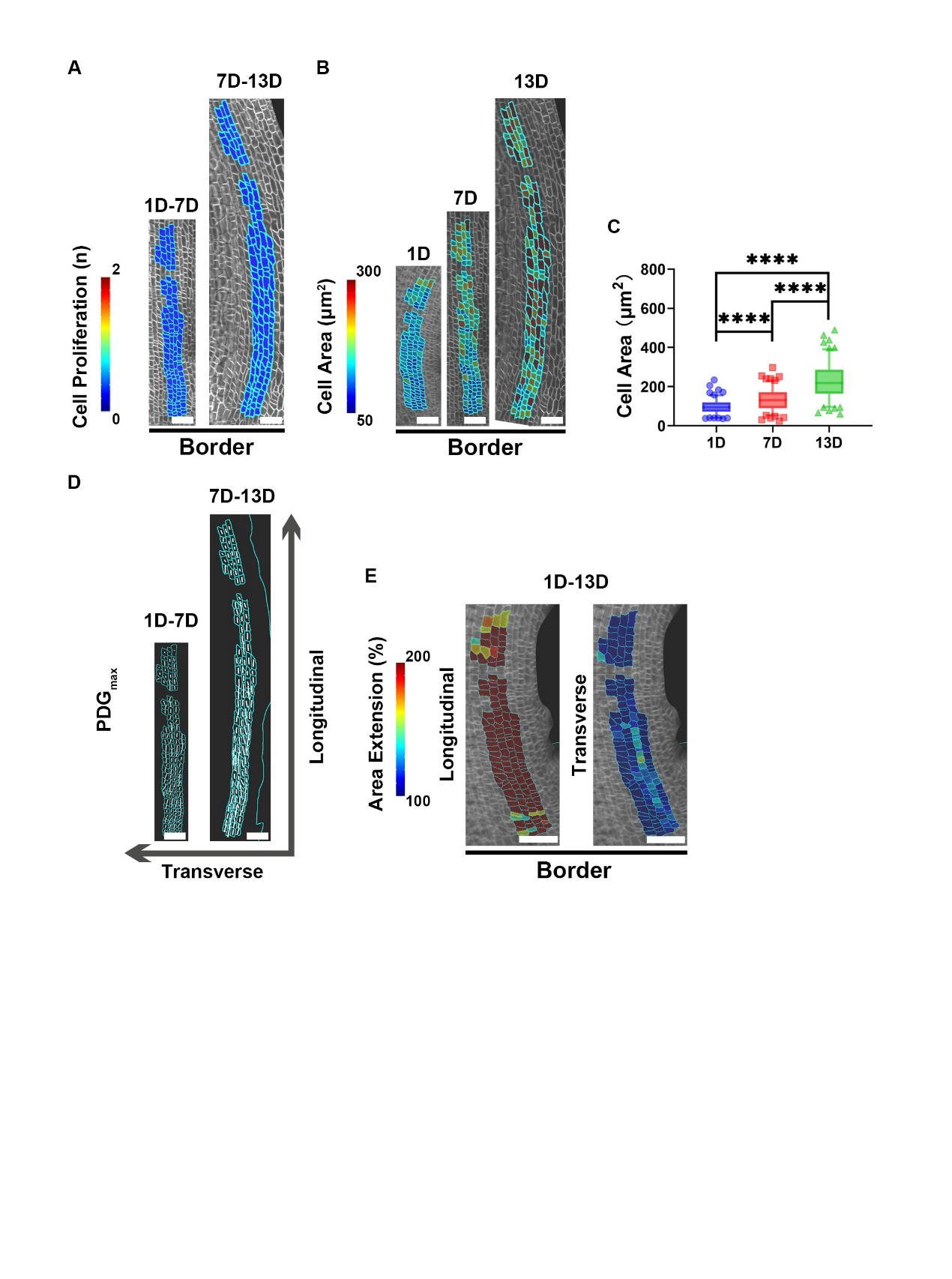


**Fig.S3 Real-time imaging of the lateral region of lamina joint epidermal cells, associated with Main Fig.2.**

**(A)** Cell proliferation in the lateral region during each growth interval (shown at the later time point). Scale bars, 50 μm.

**(B)** Heatmaps of cell area in the lateral region. Scale bars, 50 μm.

**(C)** Boxplots showing the epidermal cell area as in **(B)**. n = 134. Statistical analysis is conducted using one-way ANOVA followed by Tukey’s post-hoc test (^****^P < 0.0001).

**(D)** The directions of maximal growth (PDG_max_; PDG, principal direction of growth) for epidermal cells are indicated by white lines (shown at the later time point). Scale bars, 40 μm.

**(E)** Heatmaps of area extension (%) in the longitudinal and transverse directions from 1D to 13D of observation (shown at 1D). Scale bars, 20 μm.


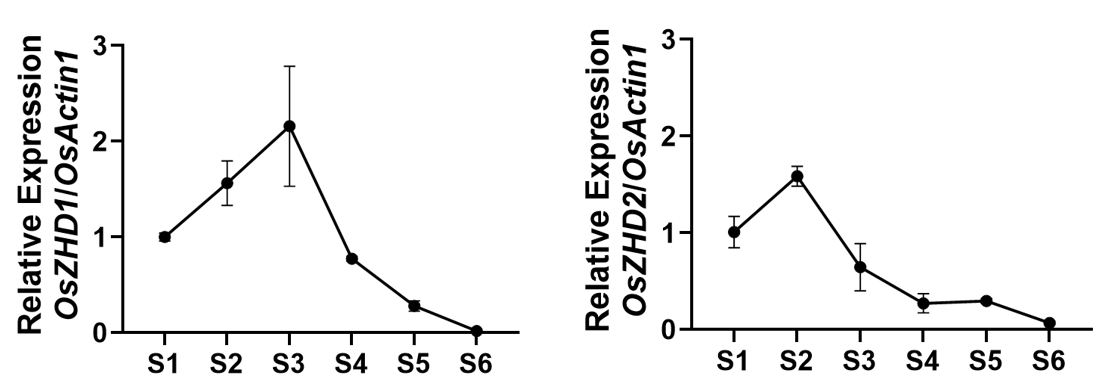


**Fig.S4 Expression patterns of *OsZHD1* and *OsZHD2*.**

*OsZHD1* and *OsZHD2* are expressed throughout lamina joint development and exhibit higher expression during the early stages (S1–S3).


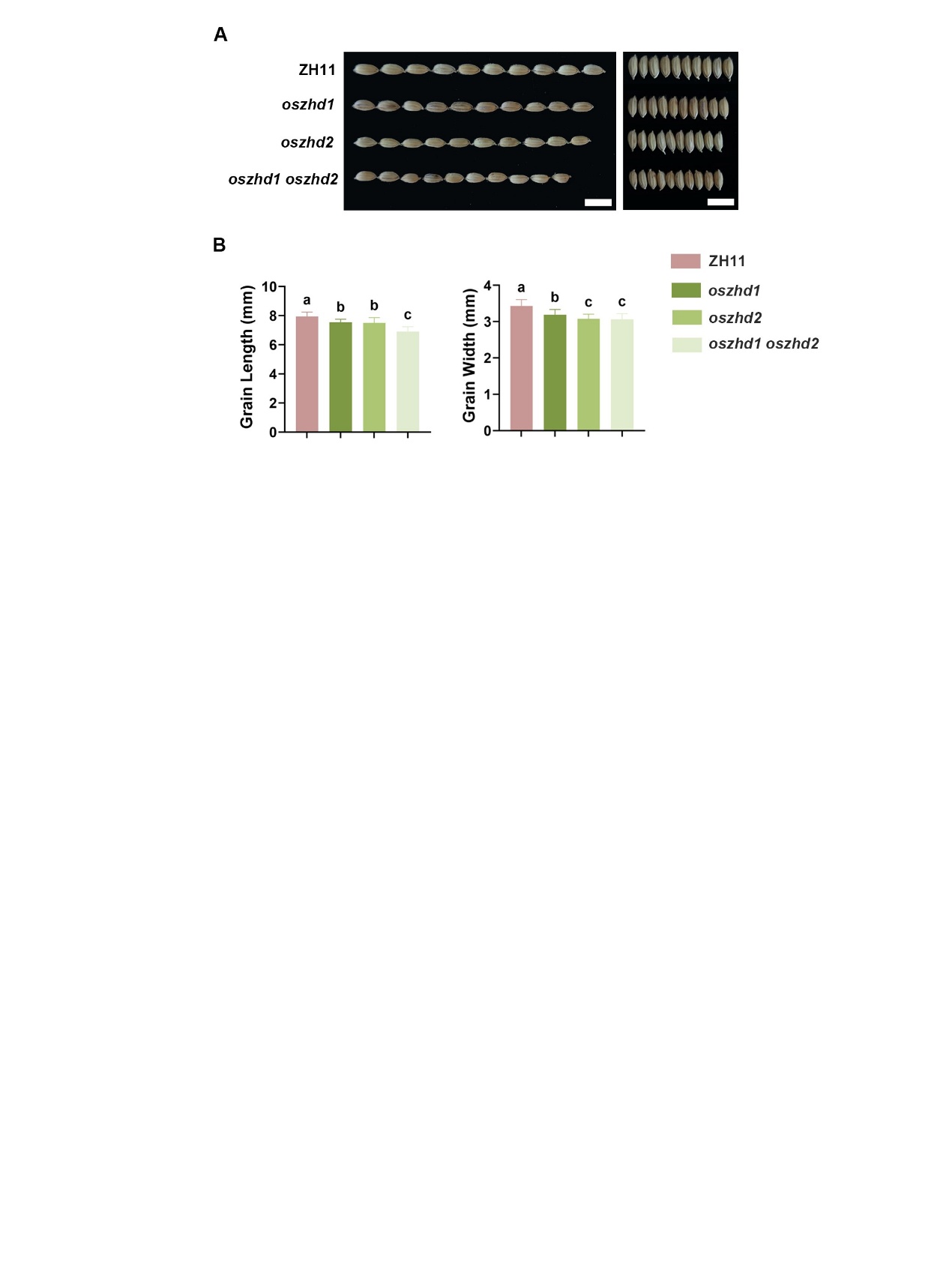


**Fig.S5 OsZHD1 and OsZHD2 knockout mutants exhibit a small grain phenotype.**

**(A)** Gross morphology of the ZH11, *oszhd1*, *oszhd2*, and *oszhd1 oszhd2* grains. Scale bars, 1 cm.

**(B)** Grain size analysis. Data are presented as mean ± SD (n = 30). Statistical analysis is conducted using one-way ANOVA followed by Tukey’s post-hoc test. Different letters indicate significant differences (P < 0.05).


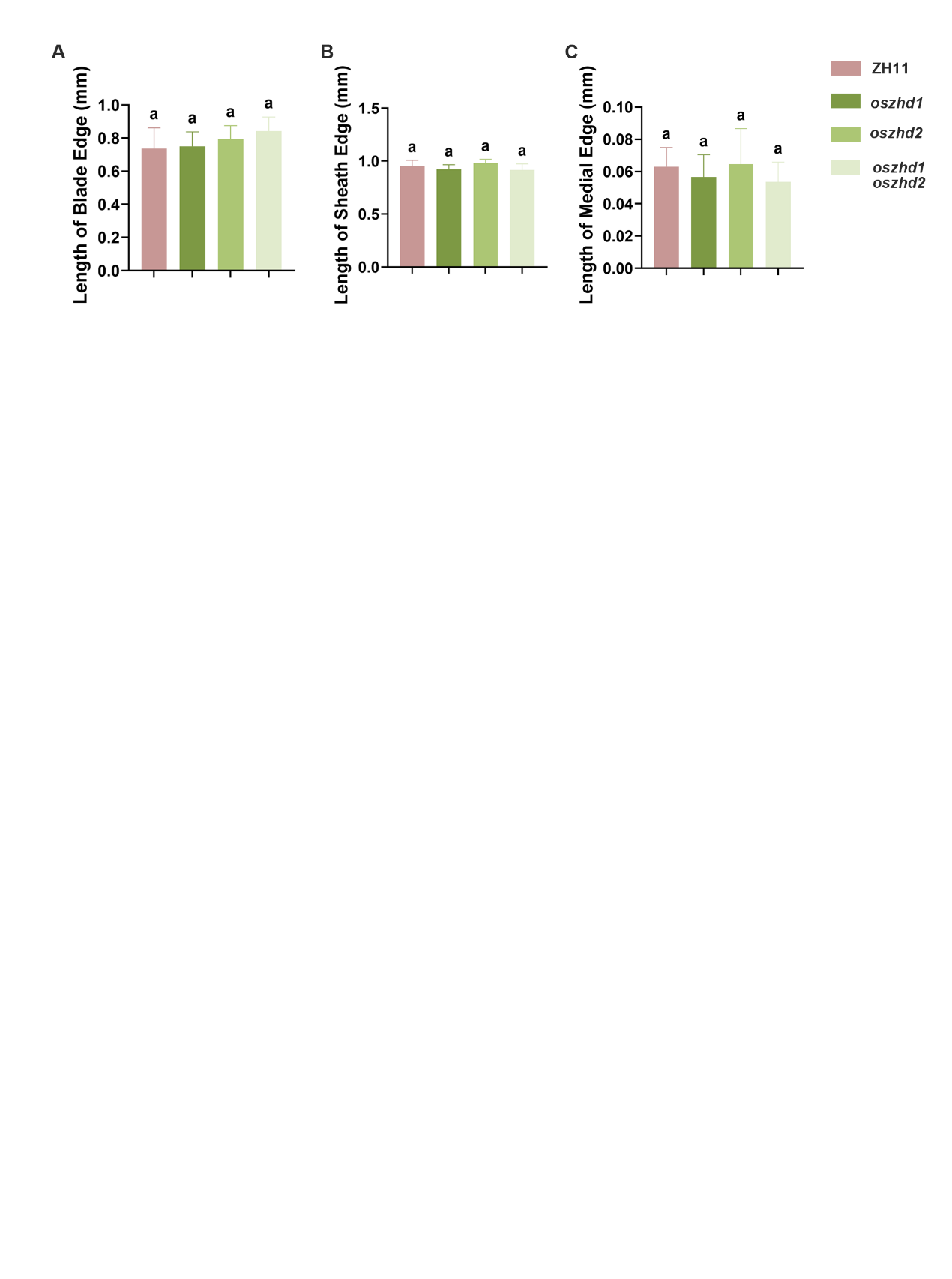


**Fig.S6 Length analysis of various knockout lines of *OsZHD1* or *OsZHD2* shown in Figure 4.**

**(A-C)** No significant differences were observed in the lengths of the blade edges **(A)**, sheath edges **(B)**, and medial edges **(C)** of the lamina joints between the different genotypes. Data are presented as mean ± SD (n ≥ 8). Statistical analysis is conducted using one-way ANOVA followed by Tukey’s post-hoc test. Identical letters indicate no significant differences (P > 0.05).


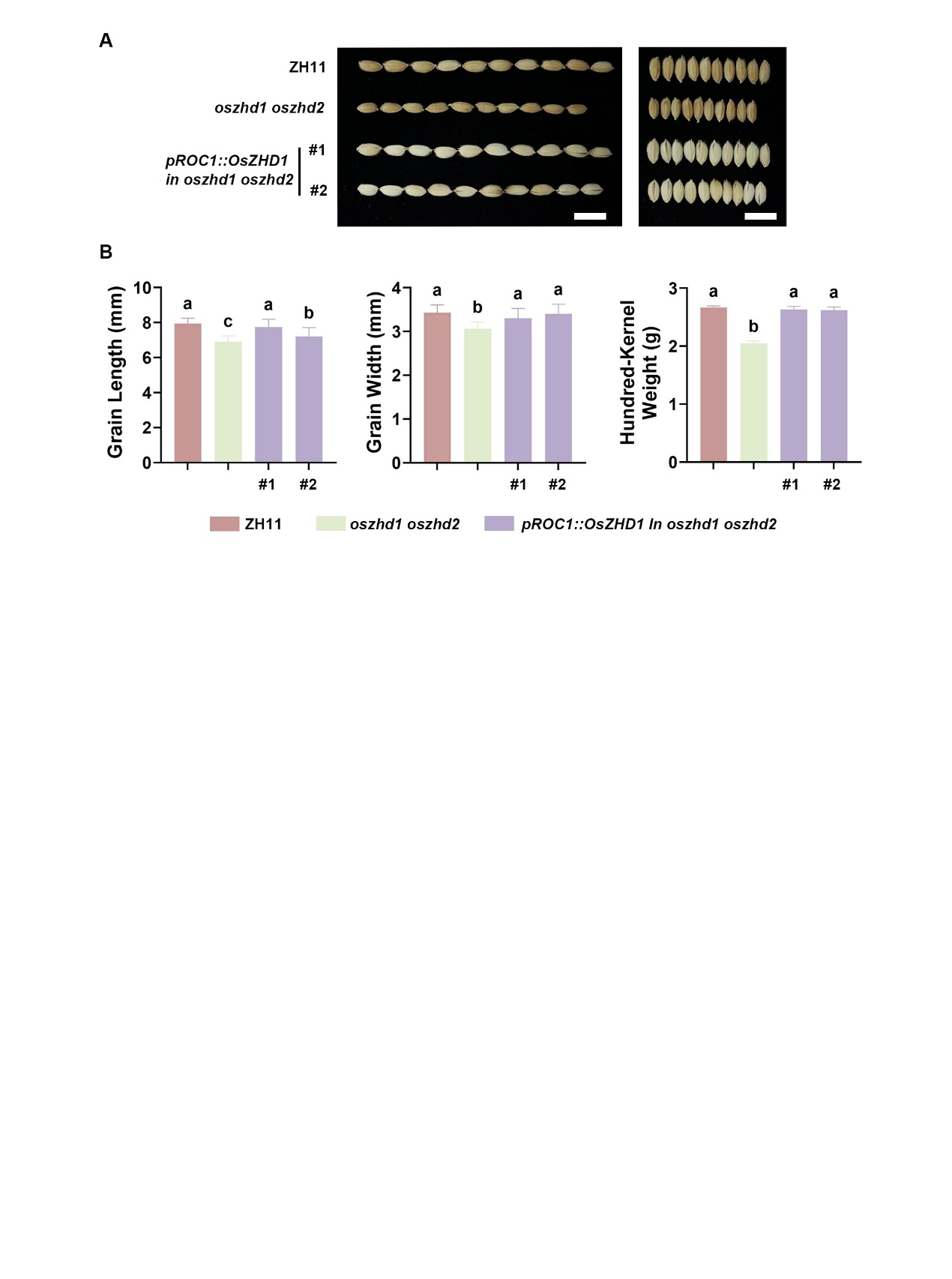


**Fig.S7 Restoring *OsZHD1* expression in the epidermis reverses the shorter grain phenotype observed in *oszhd1 oszhd2*.**

**(A)** Gross morphology of the ZH11, *oszhd1 oszhd2* and *pROC1::OsZHD1* in *oszhd1 oszhd2* grains. Scale bars, 1 cm.

**(B)** Grain size and hundred-kernel weight analysis. Data are presented as mean ± SD (n = 30 for grain size; n = 5 for hundred-kernel weight). Statistical analysis is conducted using one-way ANOVA followed by Tukey’s post-hoc test. Different letters indicate significant differences (P < 0.05).


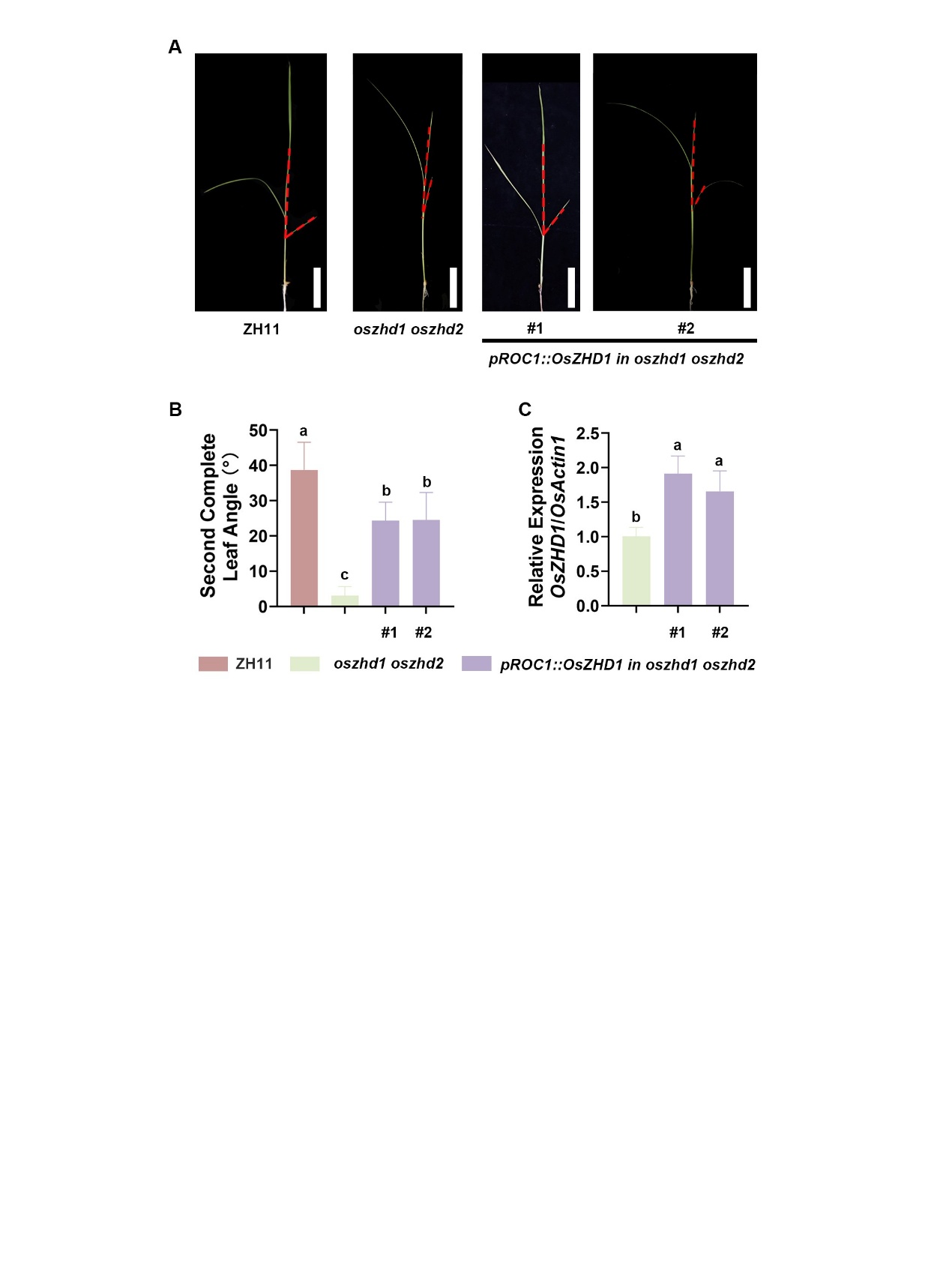


**Fig.S8 Restoring *OsZHD1* expression in the epidermis rescues the reduced leaf angle phenotype in *oszhd1 oszhd2*.**

**(A)** Gross morphology of a 4-week-old seedling. The angle between the red dashed lines represents the leaf angle. Scale bars, 5 cm.

**(B)** Measurement of the second complete leaf angle from 4-week-old seedlings. Data are presented as mean ± SD (n ≥ 9). Statistical analysis is conducted using one-way ANOVA followed by Tukey’s post-hoc test. Different letters indicate significant differences (P < 0.05).

**(C)** Transcription levels of *OsZHD1* analyzed by qRT-PCR. *OsActin1* served as an internal control for normalization. Data are presented as mean ± SD (n = 3). Statistical analysis is conducted using one-way ANOVA followed by Tukey’s post-hoc test. Different letters indicate significant differences (P < 0.05). ''#1'' refers to the transgenic line shown in Figure 5.


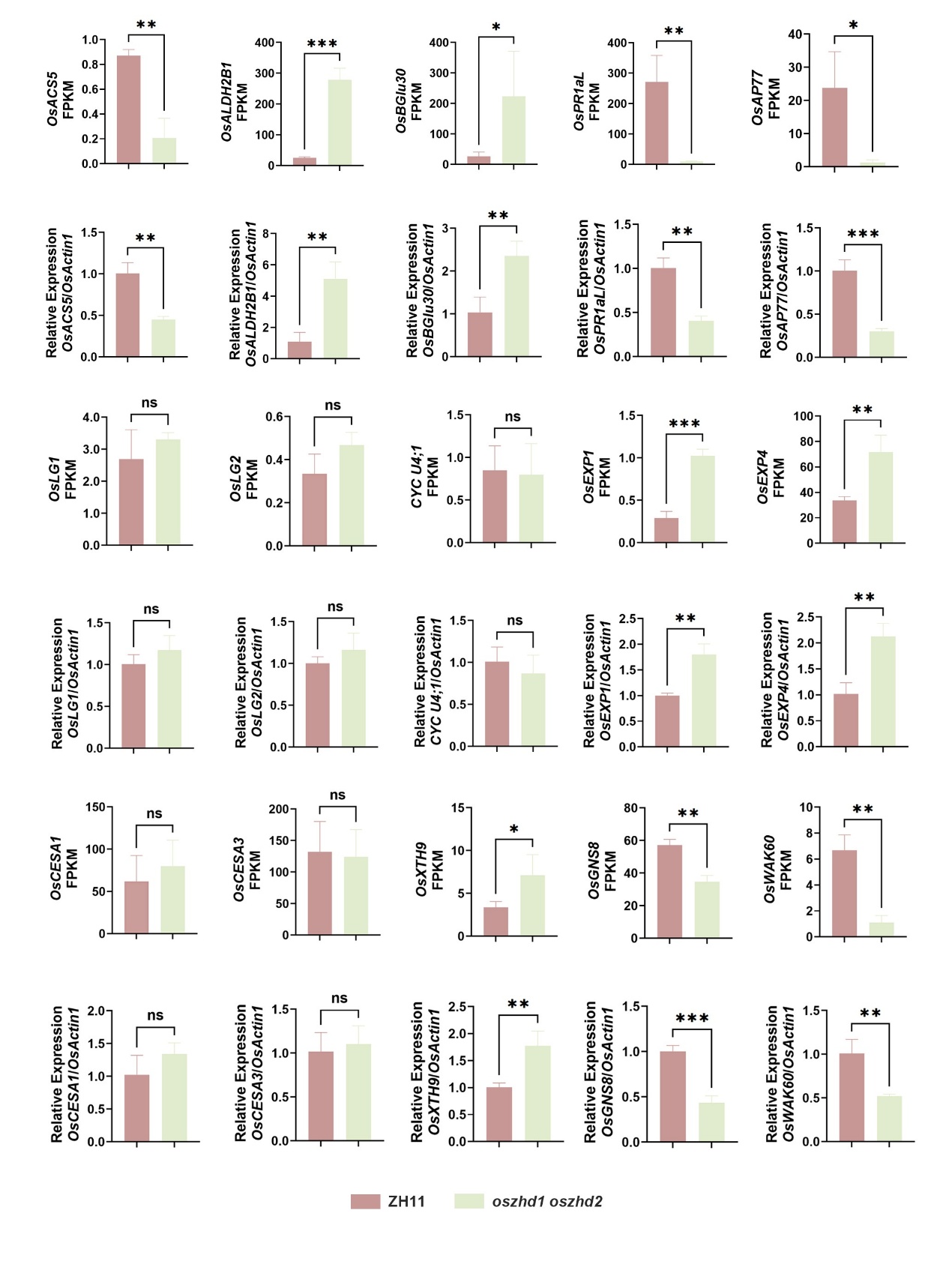


**Fig.S9** **Validation of RNA-seq results by qRT-PCR analysis.**

Expression levels of representative genes were analyzed in ZH11 and *oszhd1 oszhd2*. *OsACS5*, a known downstream target of OsZHD2, was significantly downregulated in *oszhd1 oszhd2*. *OsALDH2B1* and *OsBGlu30* (the most upregulated genes in RNA-seq) showed marked upregulation, while *OsPR1aL* and *OsAP77* (the most downregulated genes in RNA-seq) were downregulated. Early developmental stage markers (*OsLG1*, *OsLG2*, and *CYC U4;1*) exhibited no significant differences, whereas late-stage markers (*OsEXP4* and *OsWAK60*) displayed altered expression patterns. Genes associated with cell expansion and cell wall extensibility, including *OsEXP1/4*, *OsCESA1/3*, *OsXTH9*, *OsGNS8*, and *OsWAK60*, displayed expression trends consistent with the RNA-seq results: *OsCESA1/3* showed no significant change, whereas *OsEXP1/4*, *OsXTH9*, *OsGNS8*, and *OsWAK60* were significantly misregulated in the mutant. Data are presented as mean ± SD (n = 3). Statistical analysis is conducted using Student’s t-test (^*^P＜0.05, ^**^P＜0.01, ^***^P＜0.001, ns = no significance).


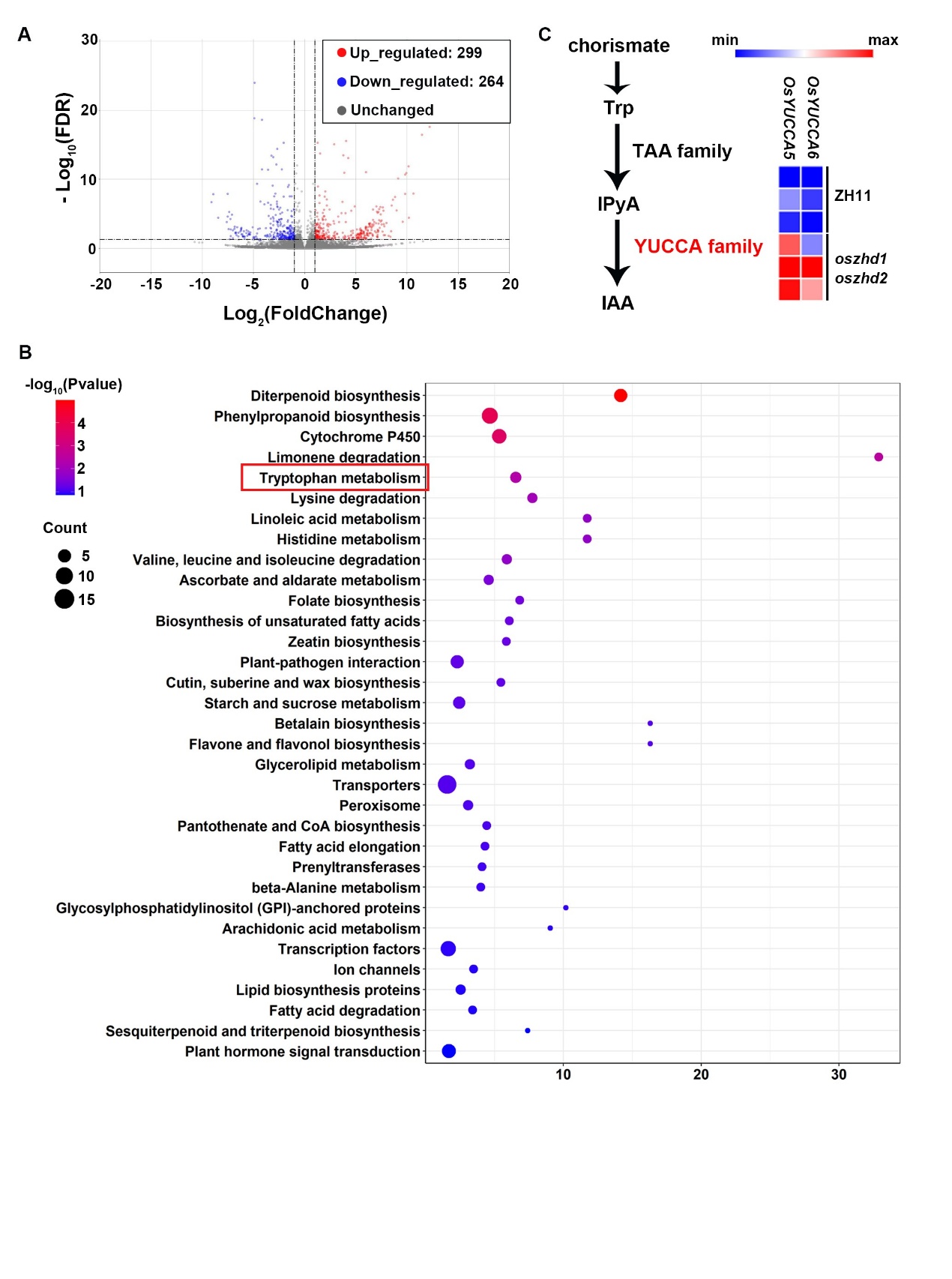


**Fig.S10 Kyoto Encyclopedia of Genes and Genome (KEGG) pathway analysis.**

**(A)** Volcano plot of differentially expressed genes (DEGs) in the mature lamina joints of 4-week-old seedlings from *oszhd1 oszhd2* compared with ZH11.

**(B)** KEGG pathway analysis categorized the DEGs in *oszhd1 oszhd2* into diterpenoid biosynthesis, phenylpropanoid biosynthesis, cytochrome P450, limonene degradation, and tryptophan metabolism.

**(C)** Tryptophan-dependent pathway for auxin biosynthesis. The YUCCA family of flavin monooxygenases catalyzes the conversion of IPyA to indole-3-acetic acid (IAA). RNA-seq analysis shows increased expression levels of *OsYUCCA5* and *OsYUCCA6*, two genes encoding enzymes for the rate-limiting step in auxin biosynthesis, in the mature lamina joint of the second complete leaf of *oszhd1 oszhd2*.


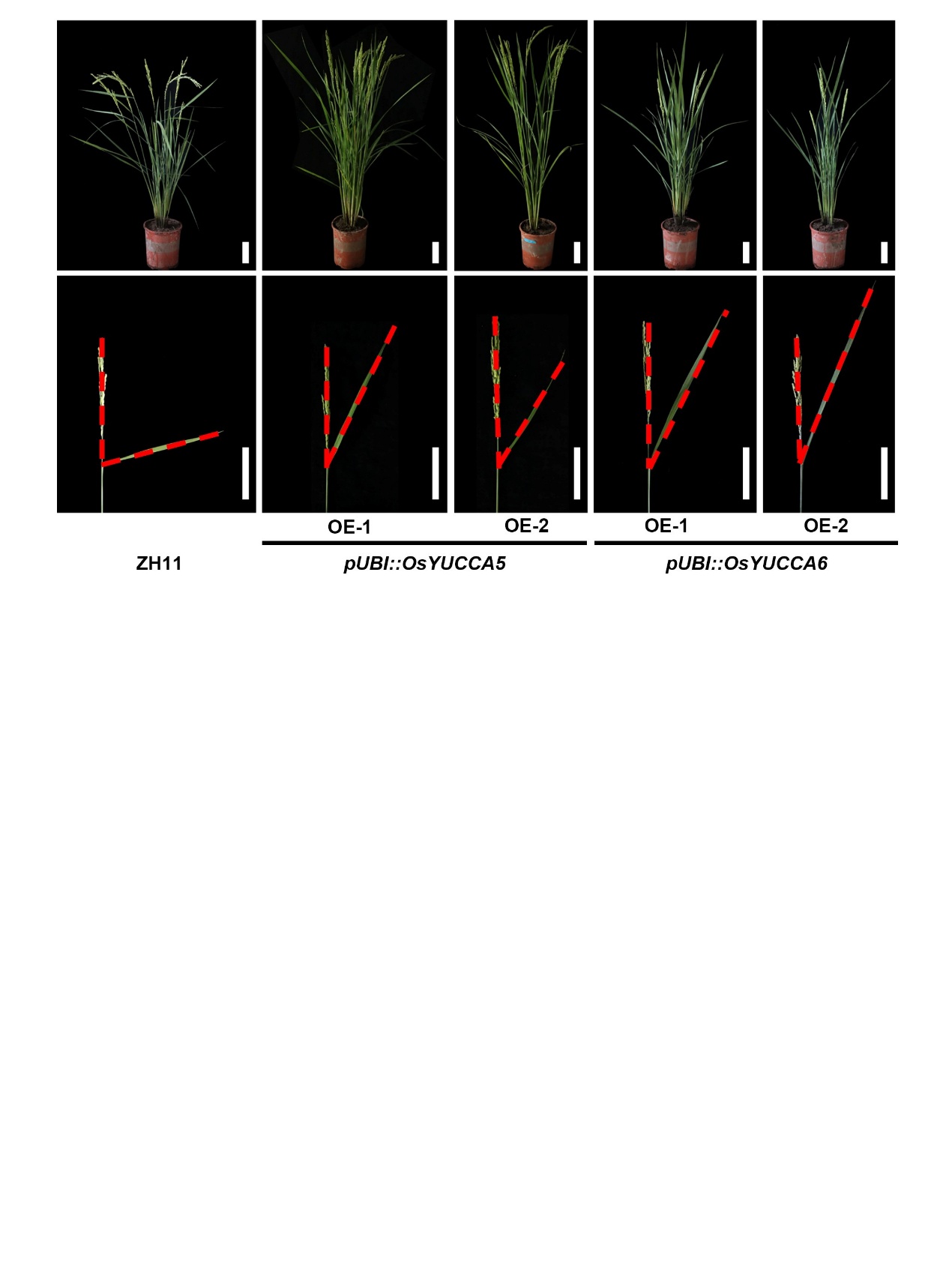


**Fig.S11 Overexpression lines of *OsYUCCA5* and *OsYUCCA6* exhibited reduced leaf angles compared to WT.**

Growth and flag leaf angle phenotypes of ZH11, *pUBI::OsYUCCA5* and *pUBI::OsYUCCA6* at the ripening stage. The red dashed lines outline the angle between the flag leaves and the stems. Scale bars, 10 cm.


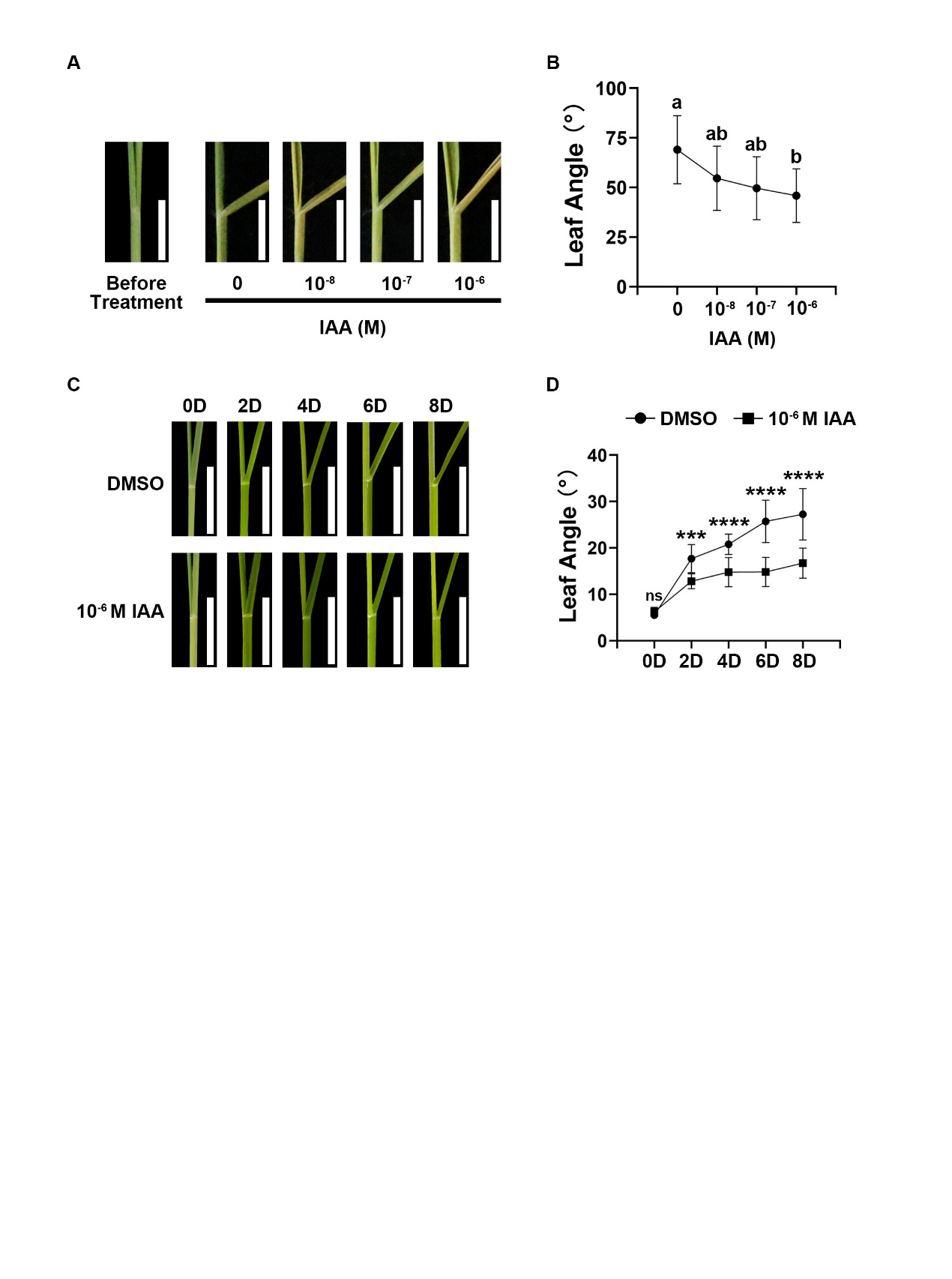


**Fig.S12 10^-6^ M IAA treatment significantly suppressed leaf angle formation.**

**(A)** Lamina joint bending response to different concentrations of IAA. Scale bars, 0.5 cm.

**(B)** Quantification of the lamina joint bending assay described in **(A)**. Data are presented as mean ± SD (n = 9). Statistical analysis is conducted using one-way ANOVA followed by Tukey’s post-hoc test. Different letters indicate significant differences (P < 0.05).

**(C)** Lamina joint bending response to 10^-6^ M IAA applied to the epidermis of the lamina joint of the second complete leaf. Scale bars, 1 cm.

**(D)** Quantification of the lamina joint bending assay described in **(C)**. Data are presented as mean ± SD (n = 11). Statistical significance is determined using Student’s t-test (^***^P < 0.001, ^****^P < 0.0001).


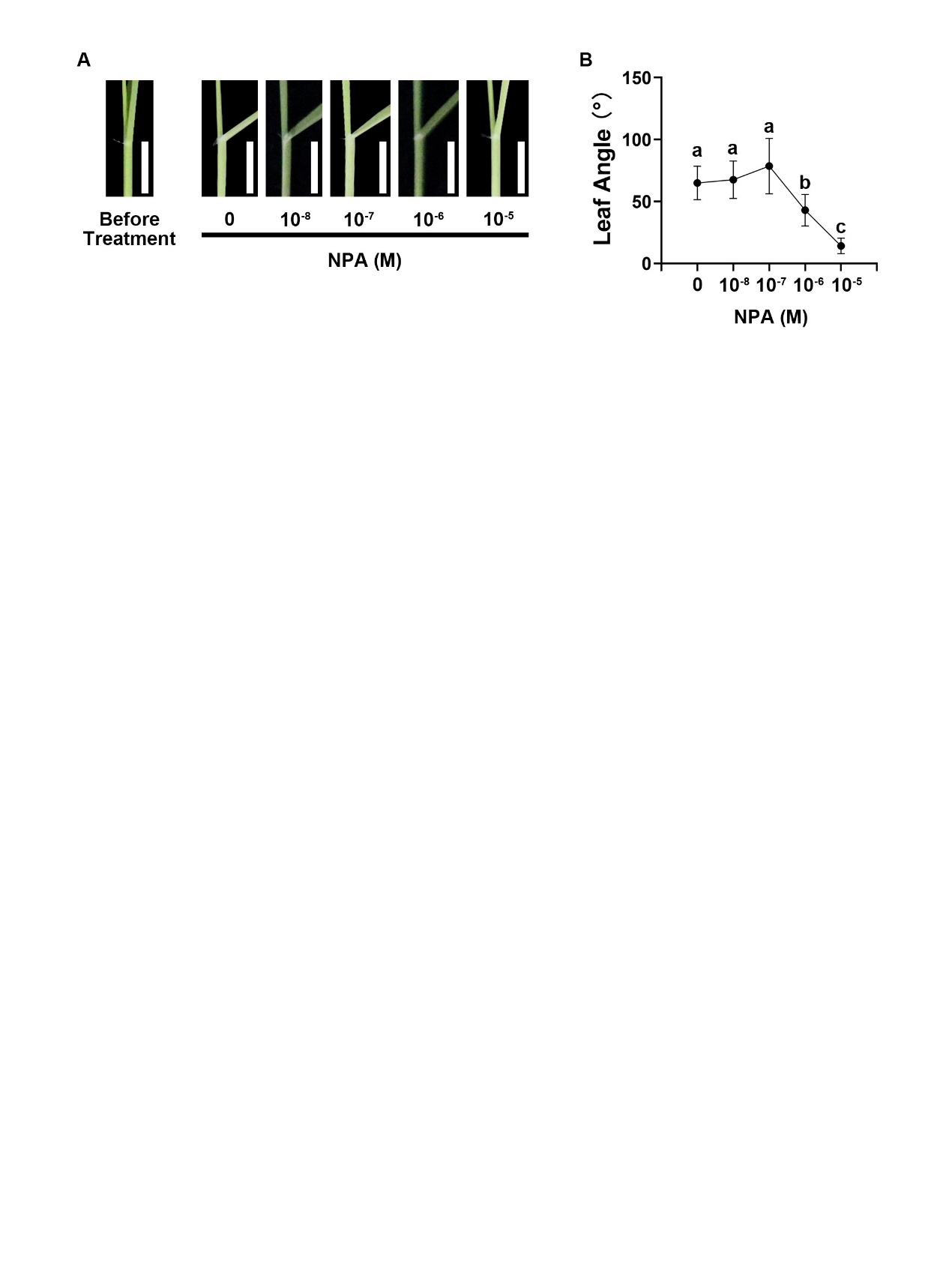


**Fig.S13 NPA treatment significantly suppressed leaf angle formation.**

**(A)** Lamina joint bending response to NPA of different concentrations. Scale bars, 0.5 cm.

**(B)** Quantification of the lamina joint bending assay described in **(A)**. Data are presented as mean ± SD (n = 9). Statistical analysis is conducted using one-way ANOVA followed by Tukey’s post-hoc test. Different letters indicate significant differences (P < 0.05).


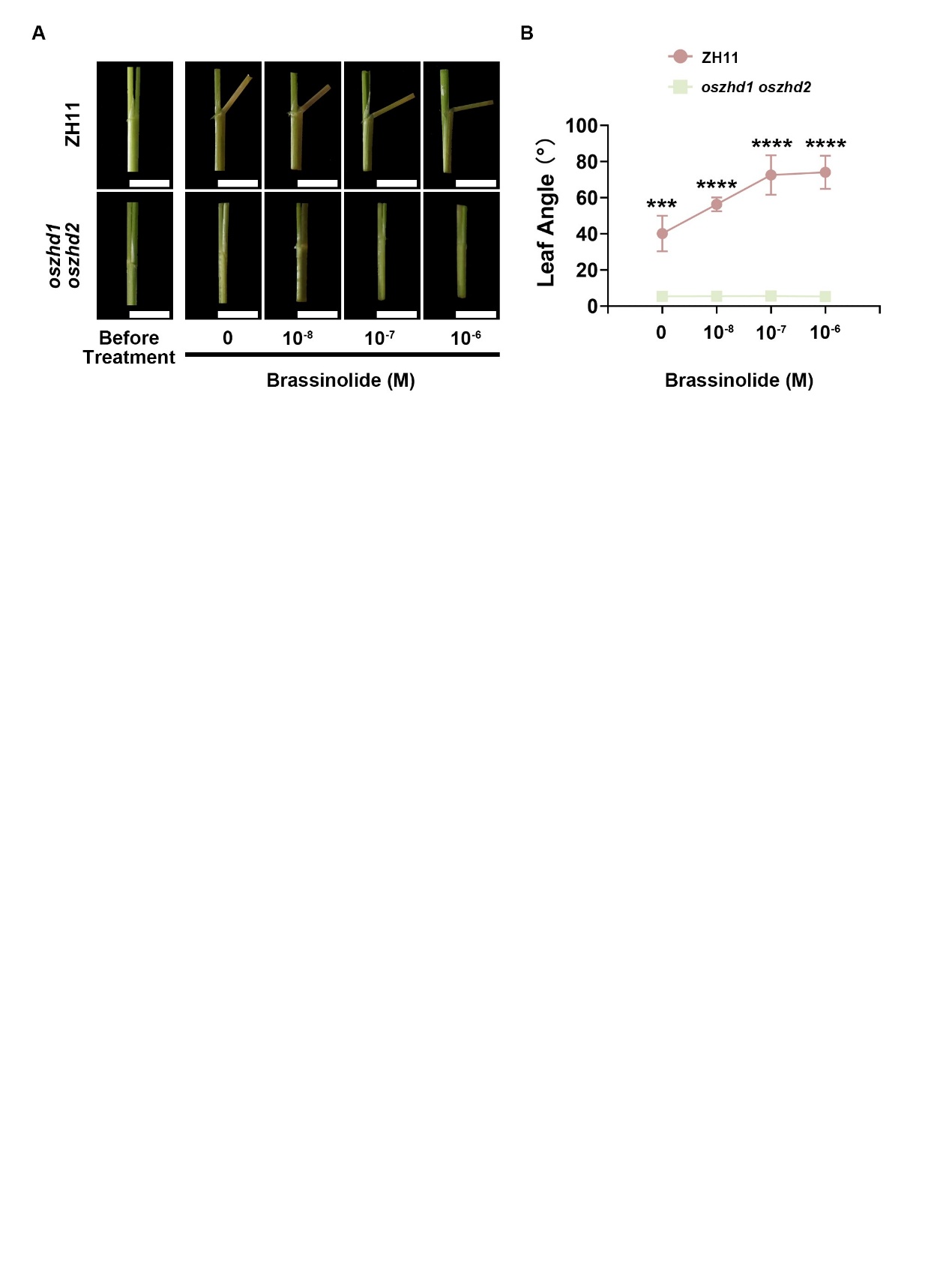


**Fig.S14 *oszhd1 oszhd2* shows reduced sensitivity to BR.**

**(A)** Lamina joint bending response to BR of different concentrations. Scale bars, 0.5 cm.

**(B)** Quantification of the lamina joint bending assay described in **(A)**. Data are presented as mean ± SD (n = 4). Statistical analysis is performed using Student’s t-test (^***^P < 0.001, ^****^P < 0.0001).


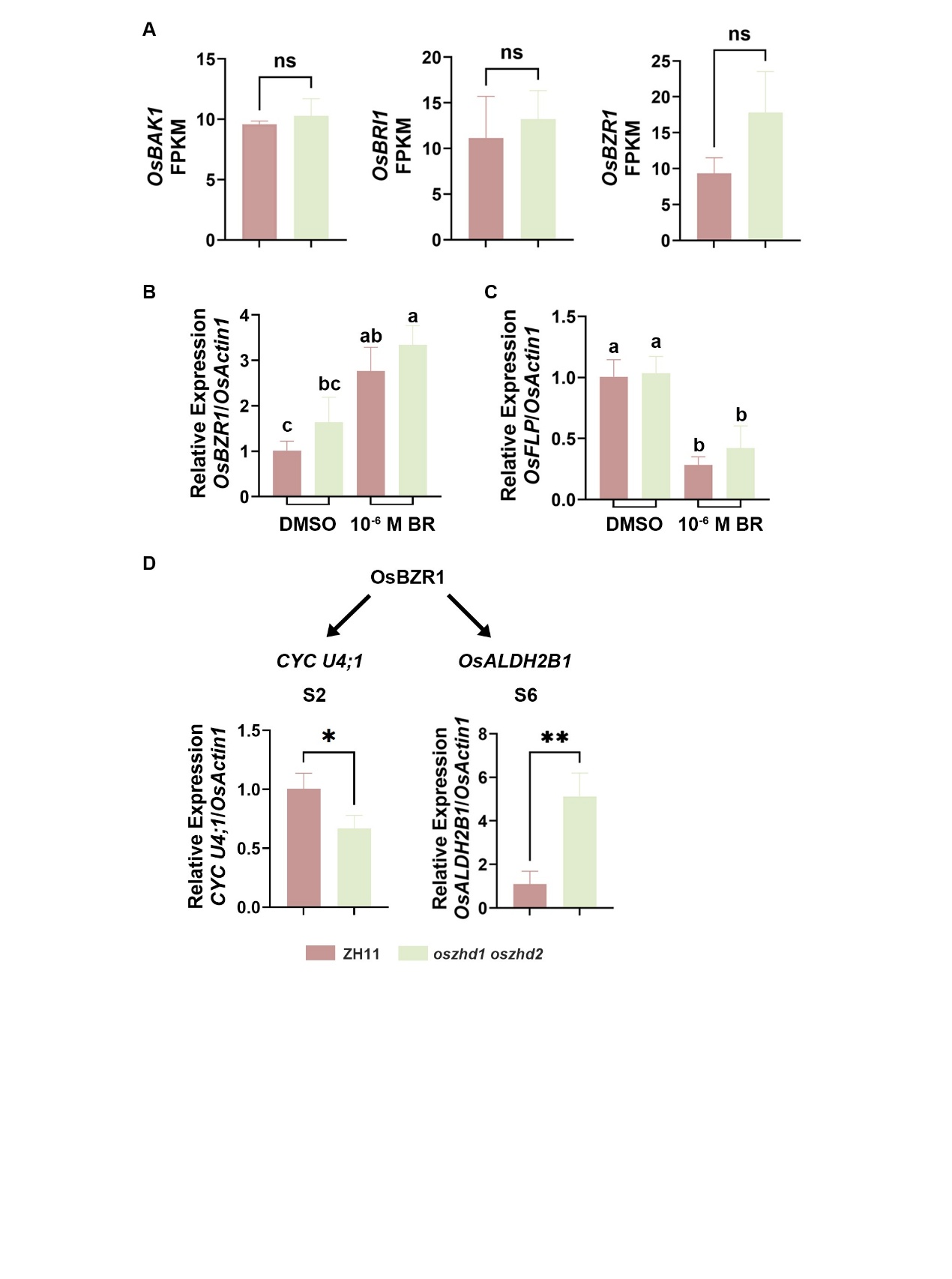


**Fig.S15 The expression of BR signaling pathway genes.**

**(A)** Expression levels of representative genes in BR signaling pathway were analyzed in ZH11 and *oszhd1 oszhd2*. Data are presented as mean ± SD (n = 3). Statistical analysis is conducted using Student’s t-test (ns = no significance).

**(B)** Expression levels of early BR signaling gene *OsBZR1*. Data are presented as mean ± SD (n = 3). Statistical analysis is conducted using one-way ANOVA followed by Tukey’s post-hoc test. Different letters indicate significant differences (P < 0.05).

**(C)** Expression levels of early BR signaling gene *OsFLP*. Data are presented as mean ± SD (n = 3). Statistical analysis is conducted using one-way ANOVA followed by Tukey’s post-hoc test. Different letters indicate significant differences (P < 0.05).

**(D)** Expression levels of *CYC U4;1* and *OsALDH2B1*, two downstream targets of OsBZR1. Data are presented as mean ± SD (n = 3). Statistical analysis is conducted using Student’s t-test (^*^P＜0.05, ^**^P＜0.01).


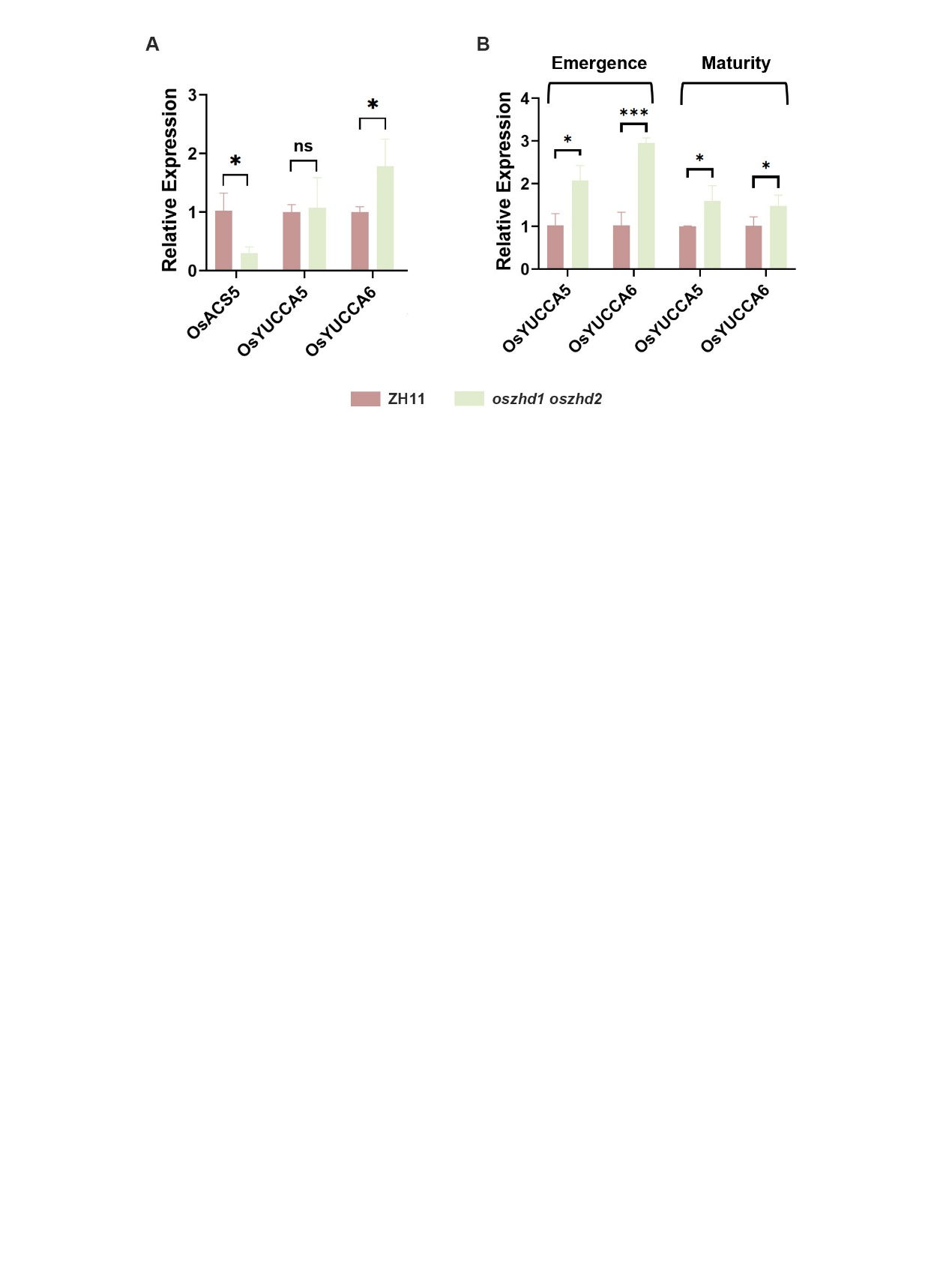


**Fig.S16 YUCCA gene expression in different tissues.**

**(A)** YUCCA gene expression in roots. Data are presented as mean ± SD (n = 3). Statistical analysis is conducted using Student’s t-test (^*^P＜0.05, ns = no significance).

**(B)** YUCCA gene expression in lamina joints at both the emergence and maturity stages. Data are presented as mean ± SD (n = 3). Statistical analysis is conducted using Student’s t-test (^*^P＜0.05, ^***^P＜0.001).


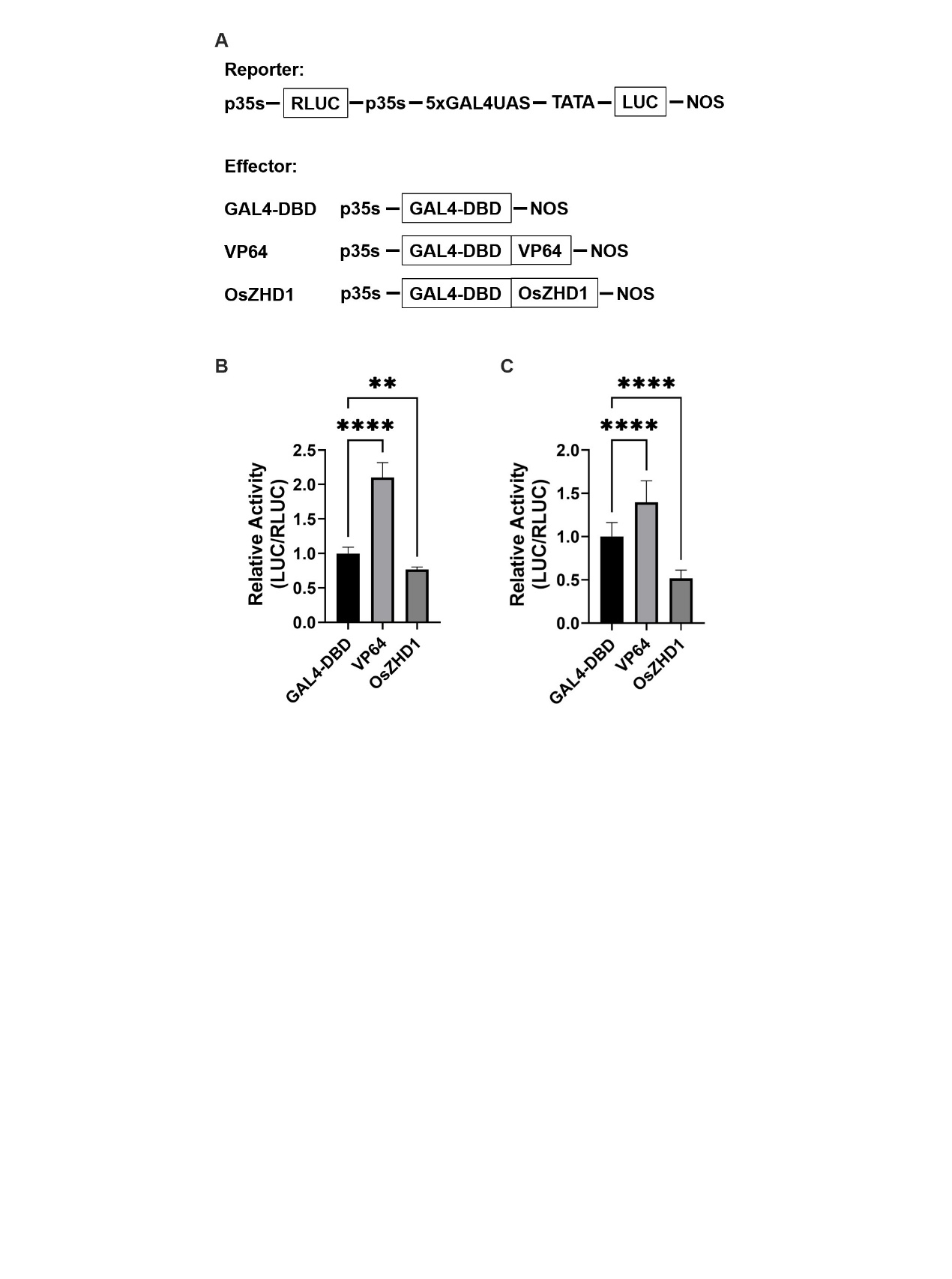


**Fig.S17 OsZHD1 exhibits transcriptional inhibition.**

**(A)** Schematic representation of constructs used in the luciferase assay.

**(B)** Relative luciferase activities measured in rice protoplast system. Data are presented as mean ± SD (n = 10). Statistical analysis is conducted using one-way ANOVA followed by Dunnett’s post-hoc test (^**^P＜0.01, ^****^P < 0.0001).

**(C)** Relative luciferase activities measured in tobacco system. Data are presented as mean ± SD (n = 7). Statistical analysis is conducted using one-way ANOVA followed by Dunnett’s post-hoc test (^****^P < 0.0001).

**
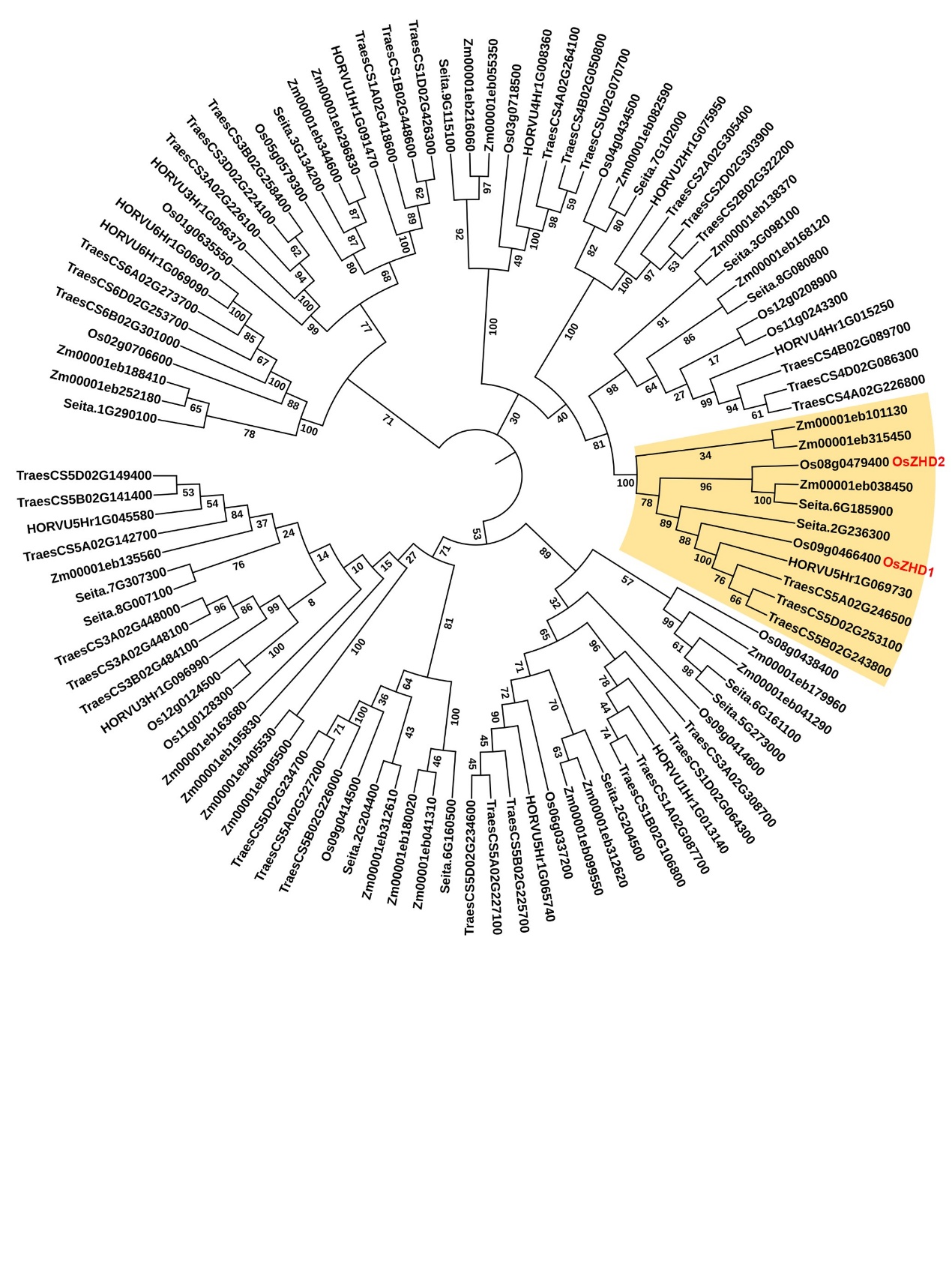
**

**Fig.S18 Phylogenetic analysis of ZF-HD proteins from rice, maize, foxtail millet, barley, and wheat.**

Protein sequences from the five representative monocot species were obtained from Phytozome v14 (https://phytozome-next.jgi.doe.gov/). Multiple sequence alignment was performed using MAFFT v7 (https://mafft.cbrc.jp/alignment/server/index.html), and the phylogenetic tree was constructed in MEGA v12 using the Neighbor-Joining (NJ) method with 1,000 bootstrap replications. A total of 103 ZF-HD proteins were identified across the five species, including 15 from rice, 24 from maize, 12 from barley, 15 from foxtail millet, and 37 from wheat. The yellow shaded region highlights the closest orthologs of OsZHD1 and OsZHD2.

**
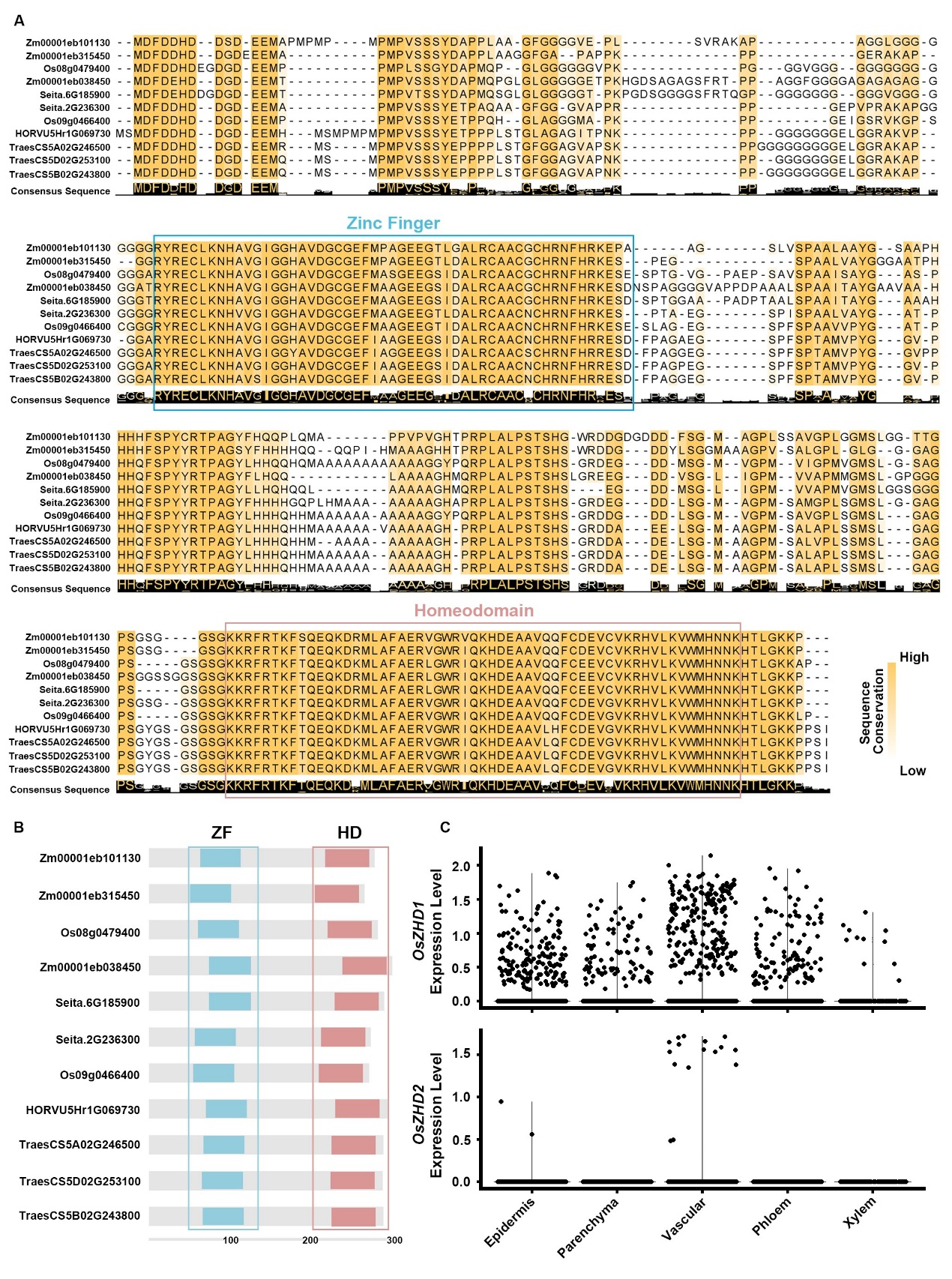
**

**Fig.S19 Conserved protein structure indicates that OsZHD1/2 potentially function in an evolutionarily conserved manner among grasses.**

**(A)** Multiple sequence alignment of OsZHD1/2 and their closest orthologs from maize, foxtail millet, barley, and wheat. Conserved residues are highlighted in orange, and the consensus sequence is shown below. The Zinc Finger and Homeodomain regions are boxed.

**(B)** Schematic representation of domain structures showing the conserved Zinc Finger (ZF) and Homeodomain (HD) regions across species.

**(C)** Single-cell expression profiles of *OsZHD1* and *OsZHD2* in different tissues of rice leaf.

**Supplemental table 1. Different genotypes of mutants.**

|  | ***OsZHD1*** | ***OsZHD2*** |
| --- | --- | --- |
| **WT** | CCAAGCCTCCTGGTGAGATTGGC | CCGCACCCCGGCAGGGTACCTCC |
|  | CCAAGCCTCCTGGTGAGATTGGC | CCGCACCCCGGCAGGGTACCTCC |
| ***oszhd1 oszhd2*#1**  **(+1+1/-2-2)** | CCAAGC(+T)CTCCTGGTGAGATTGGC | CCGCAC(-CC)CGGCAGGGTACCTCC |
|  | CCAAGC(+A)CTCCTGGTGAGATTGGC | CCGCAC(-CC)CGGCAGGGTACCTCC |
| ***oszhd1 oszhd2*#2**  **(-1+1/-1-1)** | CCAAGC(-C)TCCTGGTGAGATTGGC | CCGCAC(-C)CCGGCAGGGTACCTCC |
|  | CCAAGC(+A)CTCCTGGTGAGATTGGC | CCGCAC(-C)CCGGCAGGGTACCTCC |
| ***oszhd1 oszhd2*#3**  **(+1+1/-2-1)** | CCAAGC(+T)CTCCTGGTGAGATTGGC | CCGCAC(-CC)CGGCAGGGTACCTCC |
|  | CCAAGC(+G)CTCCTGGTGAGATTGGC | CCGCAC(-C)CCGGCAGGGTACCTCC |
| ***oszhd1 oszhd2*#4**  **(+1+1/+1+1)** | CCAAGC(+T)CTCCTGGTGAGATTGGC | CCGCAC(+A)CCCGGCAGGGTACCTCC |
|  | CCAAGC(+A)CTCCTGGTGAGATTGGC | CCGCAC(+A)CCCGGCAGGGTACCTCC |
| ***oszhd1#1***  **(+1+1)** | CCAAGC(+A)CTCCTGGTGAGATTGGC | CCGCACCCCGGCAGGGTACCTCC |
|  | CCAAGC(+A)CTCCTGGTGAGATTGGC | CCGCACCCCGGCAGGGTACCTCC |
| ***oszhd1#2***  **(+1+1)** | CCAAGC(+T)TCCTGGTGAGATTGGC | CCGCACCCCGGCAGGGTACCTCC |
|  | CCAAGC(+T)TCCTGGTGAGATTGGC | CCGCACCCCGGCAGGGTACCTCC |
| ***oszhd2#1***  **(+1+1)** | CCAAGCCTCCTGGTGAGATTGGC | CCGCAC(+A)CCCGGCAGGGTACCTCC |
|  | CCAAGCCTCCTGGTGAGATTGGC | CCGCAC(+A)CCCGGCAGGGTACCTCC |
| ***oszhd2#2***  **(-2-2)** | CCAAGCCTCCTGGTGAGATTGGC | CCGCAC(-CC)CGGCAGGGTACCTCC |
|  | CCAAGCCTCCTGGTGAGATTGGC | CCGCAC(-CC)CGGCAGGGTACCTCC |

**Supplemental table 2. Primers used in this study.**

| **Primer Name** | **Sequence** | **Purpose** |
| --- | --- | --- |
| oSX20 | TTCTGCACTAGGTACCTGCAgATGGTGAGCAAGGGCGAGGAGCTGT | F_primer for amplifing mCitrine-RCI2A fragment from *pATML1::mCitrine-RCI2A* to generate *pUBI::mCitrine-RCI2A* |
| oSX21 | TTCCCGGGGATCCGTCGACCATGAAATGATAGCGTAAGGTAT | R_primer for amplifing mCitrine-RCI2A fragment from *pATML1::mCitrine-RCI2A* to generate *pUBI::mCitrine-RCI2A* |
| oYX118 | TGCTCCTGGGTGAACTTGGT | F_primer for PCR genotyping of *OsZHD1* |
| oYX125 | GCATTTCGGCTTGACTCTT | R_primer for PCR genotyping of *OsZHD1* |
| oYX126 | TGCCACCGTAACTTCCAC | F_primer for PCR genotyping of *OsZHD2* |
| oYX120 | CGCAGACCTCCTCGCAGAA | R_primer for PCR genotyping of *OsZHD2* |
| oYX362 | gatcccccgaattactgcagAGGGTCTCTCCCTGCATCAT | F_primer for amplifing *OsROC1* fragment from WT gDNA to generate *pYX103* |
| oYX363 | ccgagctcacccggggatccGAACGAAGCCAGGTAATTAAGC | R_primer for amplifing *OsROC1* fragment from WT gDNA to generate *pYX103* |
| oYX386 | TCggatccccgggtgagctcATGGACTTCGATGACCATGACGA | F_primer for amplifing *OsZHD1* fragment from WT gDNA to generate *pOsROC1::OsZHD1* |
| oYX387 | cacttagcggccgcactagtTCATGGCAGCTTCTTGCCCAGGG | R_primer for amplifing *OsZHD1* fragment from WT gDNA to generate *pOsROC1::OsZHD1* |
| oYX202 | ttacttctgCACTAGGTACcATGGACTTCGATGACCATGACGA | F_primer for amplifing *OsZHD1* fragment from WT gDNA to generate *pUBI::OsZHD1-GR* |
| oYX190 | gtttttcgagcttcGGATCCTGGCAGCTTCTTGCCCAGGG | R_primer for amplifing *OsZHD1* fragment from WT gDNA to generate *pUBI::OsZHD1-GR* |
| oYX188 | ACcTGCAGGTCGACGGATCCgaagctcgaaaaacaaagaaaa | F_primer for amplifing *GR* fragment from *p35s::ARF3-GR* to generate *pUBI::OsZHD1-GR* |
| oYX185 | TGGCTAGCGTTAACACTAGTTCATTTTTGATGAAACAGAAGC | R_primer for amplifing *GR* fragment from *p35s::ARF3-GR* to generate *pUBI::OsZHD1-GR* |
| oYX535 | ttacttctgCACTAGGTACcATGCCAACCTCGCAACAAGATG | F_primer for amplifing *OsYUCCA5* fragment from WT cDNA to generate *pUBI::OsYUCCA5* |
| oYX536 | TGGCTAGCGTTAACACTAGTTTAATTAGCTATGTATGTCTCATGG | R_primer for amplifing *OsYUCCA5* fragment from WT cDNA to generate *pUBI::OsYUCCA5* |
| oYX547 | ttacttctgCACTAGGTACcATGGCGGCGAGGGTGGTgt | F_primer for amplifing *OsYUCCA6* fragment from WT cDNA to generate *pUBI::OsYUCCA6* |
| oYX548 | TGGCTAGCGTTAACACTAGTTCATCTTGTGGTGGCGGTTTGG | R_primer for amplifing *OsYUCCA6* fragment from WT cDNA to generate *pUBI::OsYUCCA6* |
| oYX593 | GCCACGTGCTCAAGGTGT | F_primer for *OsZHD1* qPCR |
| oYX594 | TCTCTCTTGCTACAGATTAACCT | R_primer for *OsZHD1* qPCR |
| oYX477 | AGTGGCTCAAGGGAAGTGAC | F_primer for *OsYUCCA6* qPCR |
| oYX478 | CAGCATCTGAGGAGACACCA | R_primer for *OsYUCCA6* qPCR |
| oYX479 | attcgtgaatggctgtaggg | F_primer for *OsYUCCA5* qPCR |
| oYX480 | gtcgctctcgctgaagaact | R_primer for *OsYUCCA5* qPCR |
| oYX599 | GTGTGCTTCGCCAACATGAG | F_primer for *OsACS5* qPCR |
| oYX600 | GGCAGCTAGCTTGTTGCTTT | R_primer for *OsACS5* qPCR |
| oYX583 | TGTGCTGGGTCTCGTACATTTG | F_primer for *OsALDH2B1* qPCR |
| oYX584 | TCTTGTTGAATTGCTCGTCATCA | R_primer for *OsALDH2B1* qPCR |
| oYX581 | GGTCCACAGGAATTTACACCGA | F_primer for *OsBGlu30* qPCR |
| oYX582 | GACGAACTGCAGGTGCTTTG | R_primer for *OsBGlu30* qPCR |
| oYX587 | CTATCGTACGCTGATCCGGC | F_primer for *OsPR1aL* qPCR |
| oYX588 | AGGACTAATCGATCTCACATGGT | R_primer for *OsPR1aL* qPCR |
| oYX589 | GCTTGCAGCAGGAGAACATC | F_primer for *OsAP77* qPCR |
| oYX590 | GGCCAGATCGCCACAGAATA | R_primer for *OsAP77* qPCR |
| oYX650 | CCAAGAAGAGCTGCAGGAAG | F_primer for *OsLG1* qPCR |
| oYX651 | CCATGGCTTGGTCTTGATCT | R_primer for *OsLG1* qPCR |
| oYX565 | TGGAGATCTTCCCTTCATGG | F_primer for *OsLG2* qPCR |
| oYX566 | AGGCCTGGCTTGTAGCTGTA | R_primer for *OsLG2* qPCR |
| oYX567 | CGACGACATATGCTACAACAATGC | F_primer for *CYC U4;1* qPCR |
| oYX568 | CCAAAGAGGAAGTCCACCTCAAG | R_primer for *CYC U4;1* qPCR |
| oYX698 | CAGAGCCTGTCGTTCAAGGT | F_primer for *OsEXP1* qPCR |
| oYX699 | ATAGCTCGCCACACCAAACA | R_primer for *OsEXP1* qPCR |
| oYX569 | ATAGCACGGTGTCCTTCTTCG | F_primer for *OsEXP4* qPCR |
| oYX570 | GGAAAACGTCTAAGCGGGTG | R_primer for *OsEXP4* qPCR |
| oYX700 | GTTACCTCAAAGGCGTCCGA | F_primer for *OsCESA1* qPCR |
| oYX701 | TGATATACCCGCCACCATGC | R_primer for *OsCESA1* qPCR |
| oYX702 | TGAATCATGGGGACCCCTCT | F_primer for *OsCESA3* qPCR |
| oYX703 | TTCCTTCAGCACACACCGAT | R_primer for *OsCESA3* qPCR |
| oYX706 | CAGGGTACGACCAGCAGATG | F_primer for *OsXTH9* qPCR |
| oYX707 | TCGAGTCCGCGTTCATCAAA | R_primer for *OsXTH9* qPCR |
| oYX708 | CCCGACATGACCGAAGTCTA | F_primer for *OsGNS8* qPCR |
| oYX709 | ACCGGTGACATGAGATCGAA | R_primer for *OsGNS8* qPCR |
| oYX573 | TTGATTGTTTCTGCCGTGCG | F_primer for *OsWAK60* qPCR |
| oYX574 | GCCGAATCACTTCCCATCCA | R_primer for *OsWAK60* qPCR |
| oYX597 | GTTCGAGTTCGACAAGGGGA | F_primer for *OsBZR1* qPCR |
| oYX598 | TCGAGGTCCACGAAAGGAAC | R_primer for *OsBZR1* qPCR |
| oYX648 | TGCTCAGACAACTACCCCAG | F_primer for *OsFLP* qPCR |
| oYX649 | AGAAGGCTGCTGAGAAGTGT | R_primer for *OsFLP* qPCR |
| OsActin1-F | CGGGAAATTGTGAGGGACAT | F_primer for *OsActin1* qPCR |
| OsActin1-R | AGGAAGGCTGGAAGAGGACC | R_primer for *OsActin1* qPCR |

**Supplementary table 3. Differentially expressed genes in the mature lamina joint between ZH11 and *oszhd1 oszhd2*.**
